# A Pilot Graduate Student-Led Near-Peer Mentorship Program for Transfer Students Provides a Supportive Network at an R1 Institution

**DOI:** 10.1021/acs.jchemed.2c00427

**Published:** 2022-11-10

**Authors:** Audrey G. Reeves, Amanda J. Bischoff, Brice Yates, Daniel D. Brauer, Anne M. Baranger

**Affiliations:** † Department of Chemistry, University of California, Berkeley, California 94720, United States; § Molecular Biophysics and Integrated Bioimaging Division, Lawrence Berkeley National Laboratories, Berkeley, California 94720, United States; ‡ Graduate Group in Science and Mathematics Education, University of California, Berkeley, California 94720, United States

**Keywords:** Graduate Education/Research, History/Philosophy, Collaborative/Cooperative Learning, Enrichment/Review Materials, Student/Career Counseling, Testing/Assessment, Undergraduate Research, Minorities in Chemistry

## Abstract

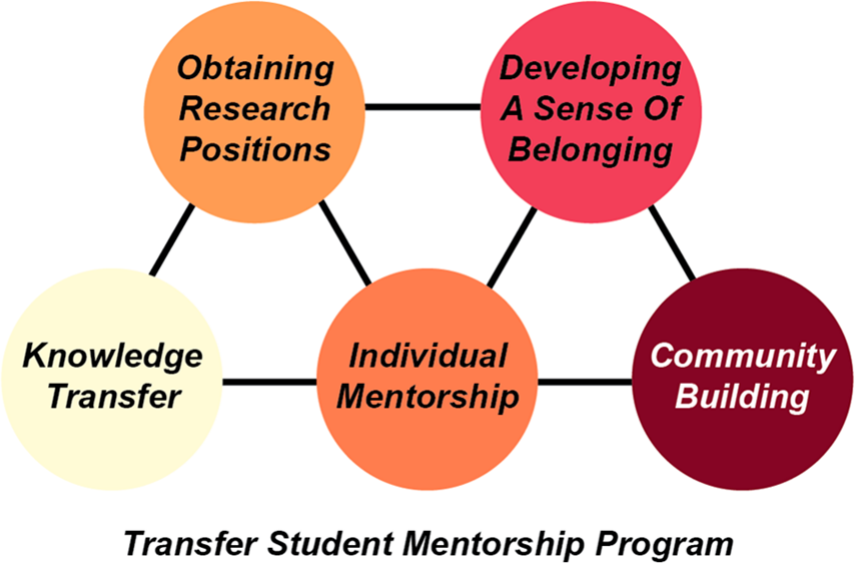

The undergraduate transfer process has well-documented challenges, especially for those who identify with groups historically excluded from science, technology, engineering, and mathematics (STEM) programs. Because transfer students gain later access to university networking and research opportunities than first-time-in-college students, transfer students interested in pursuing postbaccalaureate degrees in chemistry have a significantly shortened timeline in which to conduct research, a crucial component in graduate school applications. Mentorship programs have previously been instituted as effective platforms for the transfer of community cultural wealth within large institutions. We report here the design, institution, and assessment of a near-peer mentorship program for transfer students, the Transfer Student Mentorship Program (TSMP). Founded in 2020 by graduate students, the TSMP pairs incoming undergraduate transfer students with current graduate students for personalized mentorship and conducts discussion-based seminars to foster peer relationships. The transfer student participants have access to a fast-tracked networking method during their first transfer semester that can serve as a route for acquiring undergraduate research positions. Program efficacy was assessed via surveys investigating the rates of research participation and sense of belonging of transfer students. We observed that respondents that participated in the program experienced an overall improvement in these measures compared to respondents who did not. Having been entirely designed, instituted, and led by graduate students, we anticipate that this program will be highly tractable to other universities looking for actionable methods to improve their students’ persistence in pursuing STEM degrees.

## Introduction

Transferring from a community college to a four-year university provides a feasible route to an advanced degree for many students, primarily due to its affordability.^1−3^ In addition to entering a social sphere on average two years after first-time-in-college (FTIC) students, transfer students are disproportionally members of historically excluded groups from Science, Technology, Engineering, and Mathematics (STEM).^4^ Many transfer students also experience transfer shock, a decrease in academic performance after transferring to a baccalaureate-granting institution. The detrimental effects of transfer shock can be mitigated when students have a strong sense of community at their new institution.^5^ Accelerating the process of forging connections between transfer students and existing students and faculty at their transfer institution provides a direct, actionable method for improving equity and inclusion in the sphere of STEM academia.

College students’ persistence in STEM is enhanced by mentoring, advising, participating in research, participating in bridge programs, and many other factors.^6^ Programs combining multiple approaches to facilitating persistence in STEM have beneficial outcomes for a larger proportion of students than programs with a single component.^7^ For undergraduate transfer students, mentoring by peers and faculty can enhance enculturation into the university setting.^8,9^ Near-peer mentoring of undergraduates by more senior undergraduates or graduate students has also been demonstrated to enhance persistence in STEM and science identity.^10,11^ These mentoring relationships have demonstrated positive effects on the science identity, self-efficacy, and sense of belonging of the mentors involved as well as mentees.^12^

Undergraduate research experience is critical for advancing through higher education in STEM.^13,14^ Strong connections have been made between undergraduate research experience and an improved GPA or the decision to continue to an advanced degree.^15^ This trend is augmented among undergraduates that identify with groups that have been historically excluded from STEM fields.^16−18^ The persistence of community college and transfer students in STEM has also been enhanced through participation in undergraduate research experiences.^19^ Formal instruction on how to apply for undergraduate research positions is limited or nonexistent at many institutions, and many principal investigators (PIs) find undergraduate students to fill these positions via networking within the institution.^20^ Furthermore, research experience and a letter of recommendation from a supervising PI are highly beneficial in applications for postgraduate degrees.^21^

Through the graduate student-led development of the Transfer Student Mentorship Program (TSMP), we aimed to provide transfer students with information about undergraduate research and postgraduate degrees, build community among transfer students, and provide mentorship before and during the first transfer semester. This program would entail one-on-one mentorship of transfer students by graduate students and periodic group seminars and discussions on research-based topics with graduate and transfer student mentors. We sought to (1) create a voluntary, near-peer mentorship program and understand its participation level and effect on sense of belonging and research participation, and (2) provide a framework for the setup of similar programs for transfer students at comparable institutions with a heavy research focus but low faculty to undergraduate student ratio. We also aimed to understand whether this program would increase research participation among transfer students, and whether it would result in changes in the sense of belonging among both transfer students and graduate student mentors.

## Theoretical Framework

Community cultural wealth (CCW) theory postulates that sources of capital outside of monetary capital such as navigational, social, and familial capital can be beneficial in navigating institutions for underrepresented communities within larger power structures.^22^ In an academic sphere, an undergraduate student with high CCW might have a parent or sibling in STEM, whereas a student with low CCW might be the first in their family to attend college. Students with higher cultural capital as measured by familiarity with “rules of research” have been demonstrated to have greater success securing STEM undergraduate research positions.^20^ One method that has been previously assessed as effective in increasing these sources of capital and communicating the hidden curriculum instrumental to educational and career progression within an academic sphere is mentorship.^23−26^ In an academic context, deliberate pairing of new students (low capital) with established mentors (high capital) initiates the flow of community wealth downstream, providing a clear path to increase access to continuing higher education.

Underrepresented communities’ entrance and persistence in STEM also connects to social identity theory (SIT), which asserts that a portion of an individual’s sense of self derives from perceived membership in a relevant social group.^27,28^ The persistence of students from underrepresented groups in STEM has been correlated with higher science identity.^17^ In the context of mentorship, SIT offers a connection for an individual who perceives themselves in the “out group”, often a result of historical exclusion by an institution, with someone they perceive as being in the “in group”, a person who is already established in this community. With this connection, the individual can more easily visualize the transfer of themselves into the “in group”. In this work, the “in group” is generally defined as those in academic and/or industrial positions of power with high capital.

Where CCW highlights the transition of an individual more literally to a status of higher capital, SIT reflects how this individual perceives themselves as *belonging* in this group of higher capital. Together, the frameworks of CCW and SIT provide a lens to understand how a mentorship-centric program such as the TSMP may improve the transfer student experience within a STEM discipline.

## Program Description and Outcomes

### Objectives

**Figure 1:**
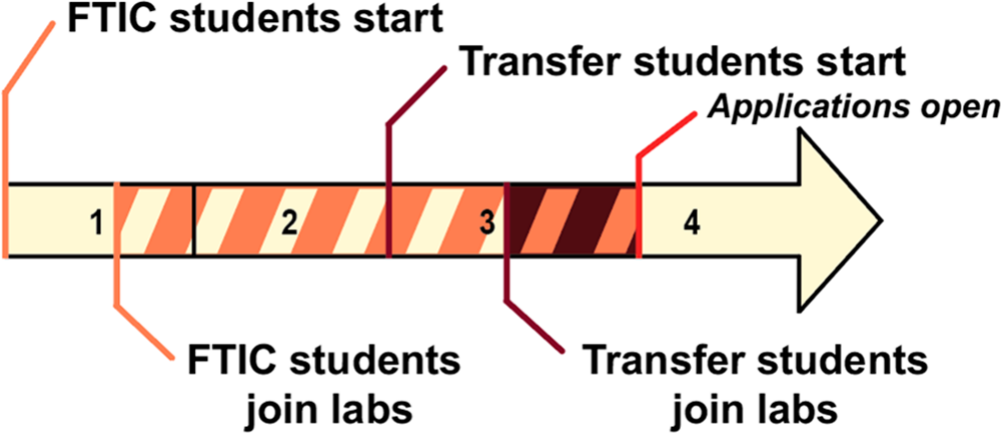
A timeline comparison between a first-time-in-college student (FTIC) and transfer student that matriculated at the beginning of their junior year, where both find undergraduate research positions as a result of a connection made in the classroom, both graduate in four years, and both are interested in attending graduate school immediately after graduation. Under these circumstances, the FTIC student has 2.5 years of performing research before preparing graduate school applications (orange dashed), while the transfer student only has one semester and summer (maroon dashed).

Transfer students frequently have a shorter timeline for establishing professional connections and participating in research than FTIC students. A timeline comparing this difference between a transfer student that matriculated at the beginning of their junior year and an FTIC student both interested in pursuing advanced degrees immediately after graduation is provided in Figure 1. This timeline outlines one example of how a student might find a research position through a connection made in a classroom setting. In this timeline, it is assumed that both students successfully acquire an undergraduate research position by the conclusion of their first semester.

Undergraduate research positions vary widely in their admissions processes, work styles, and availabilities, even within a single department, and there are no clear routes as to how to best obtain such positions. These positions are largely filled via informal networking, frequently a result of a graduate student meeting an interested undergraduate in a course they’re teaching.^20^ These factors place transfer students interested in performing undergraduate research at a significant disadvantage when applying to such positions. For a transfer student intending to apply for graduate programs during their senior year, even an ideal situation such as that outlined in Figure 1 would leave them with only one semester and one summer in a research lab before the graduate school application cycle opens.

The programmatic goals of the TSMP were 3-fold: (1) to instruct transfer students on aspects of the hidden curriculum present in academic culture, (2) to provide fast-tracked networking to place interested transfer students in undergraduate research positions, and (3) to provide a community for transfer students as they entered the university. Our three goals are reflected in the design of the TSMP, which consists of group seminars, one-on-one mentorship meetings with graduate student mentors, and small-group discussions involving senior transfer student mentors, all taking place during the transfer students’ first transfer semester. Compared to a classroom setting where relationships with peers, graduate teaching assistants, and professors would typically be established over the course of a semester, these near-peer mentors can extend their personal network to their transfer student mentees based on their research interests as early as their first meeting at the beginning of their transfer semester.

### Leadership and Participant Recruitment

The TSMP was designed entirely by graduate students and led by two codirectors, both graduate students. The codirectors were responsible for designing and presenting seminars, reserving venues for in-person meetings, matching graduate student mentors with transfer students, and coordinating graduate student mentors and transfer student mentors. Codirectors also provided guidance regarding mentorship topics and distributed information on open positions in research laboratories to the program. Transfer student mentors supported various administrative tasks under the supervision of the codirectors, which included sending emails regarding upcoming seminars to all transfer students, ordering and distributing food at seminars, coordinating the virtual room held concurrently with the in-person seminars, and guiding small-group discussions.

Recruitment of undergraduate participants began immediately after students were admitted to the university in April. At the public R1 institution and during the year studied, transfer students made up 16% of the undergraduate population of the Departments of Chemistry and Chemical and Biomolecular Engineering. The program was advertised to all junior and senior transfer students via email at multiple points throughout the summer before commencement of the program. The program was first advertised as part of a panel on programs available for transfer students, held during a recruitment weekend and open to all recently accepted transfer students. The main source of advertisement to the TSMP was through an email sent to all incoming transfer students to the Departments of Chemistry and Chemical and Biomolecular Engineering, describing the program and including a form for interested parties to sign up. This led to 35 junior transfer students and eight senior transfer students signing up for participation in the TSMP (39% of all junior transfer students and 13% of all senior transfer students). No application process was required; thus, all students who signed up were able to participate in the TSMP. All junior TSMP participants were matched with a graduate student mentor.

Graduate and transfer student mentors were also recruited via email. The TSMP was advertised to all graduate students in the Department of Chemistry via a weekly email sent out to the entire department for the duration of a month the summer before the TSMP began. The email briefly described the program and linked to a form where interested students could sign up to become a mentor. In this manner, we successfully recruited 20 graduate student mentors and paired each with either one or two mentees. Transfer student mentors were recruited from the previous year’s program class of transfer students. Transfer students in the 2020 TSMP cohort were emailed and asked to fill out a short application if they had an interest in providing mentorship for the new class of transfer students. In this manner, we successfully recruited four transfer student mentors.

### Program Description

**Figure 2:**
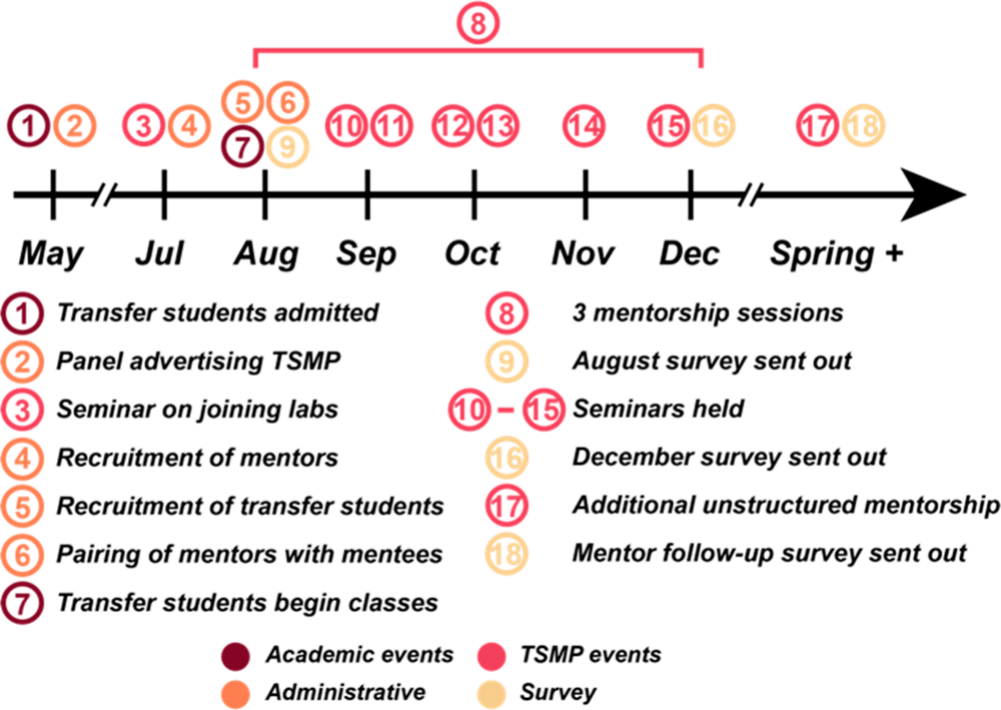
Timeline of administrative and programmatic events for the TSMP alongside academic and survey timelines.

The TSMP began with a seminar open to all incoming transfer students on the subject “Finding an undergraduate research position” one month before their first transfer semester. This seminar was given both with the intention of recruiting students to the program and to help them understand how to approach obtaining a research position in an academic lab before they started their first semester at Berkeley. Following this seminar, transfer students and mentors were recruited. A 1 h introductory meeting and mentorship training were required for graduate and transfer student mentors before the program began, led by the program codirectors. Transfer students were matched to an appropriate mentor both by subfield of interest and specific identity when requested and possible (e.g., mentors that matched the student’s gender or race, or who also identified as first-generation college students). Mentors were matched with a maximum of two mentees and instructed to set up 3–5 meetings with their mentees over the course of the fall semester. Graduate student mentors were exclusively members of the chemistry graduate program, while transfer students were split evenly between Chemistry and Chemical and Biomolecular Engineering majors. A general timeline of the fall semester program scheduling is provided in Figure 2.

During the fall semester, the TSMP programming consisted of both one-on-one meetings between graduate student mentors and transfer students, and discussion-based small-group seminars. Group discussions were divided into thirds: beginning with an unstructured social period over a provided dinner, followed by the indicated seminar presented by one of the codirectors (or a transfer student mentor on one occasion), and concluding with small-group discussions led by a combination of graduate student mentors and transfer student mentors. The discussion portion provided a valuable source of structured peer-to-peer mentorship between incoming transfer students and senior transfer student mentors. While these discussions were optional for all transfer student participants, all transfer student mentors and at least six graduate student mentors were required to attend each discussion. The topics for the small-group seminars are detailed in Table 1.

**Table 1:** Seminar Topics Covered in TSMP Meetings in Fall 2021 and Their Descriptions

Seminar Topics	Description
Applying for Research Positions	How to contact research groups for the greatest chance of acquiring an undergraduate research position
Navigating Classes	Advice on building a schedule most efficiently; led by a senior transfer student
Introduction to Research Groups	Description of various research groups in the Departments of Chemistry and Chemical and Biomolecular Engineering; choosing which subdiscipline is best for you
Careers in Chemistry	Career paths available with a bachelor’s, master’s, or doctorate degree in chemistry
Applying for Funded Research Opportunities	How to apply for external funding in graduate programs

Seminar topics were selected to communicate aspects of the hidden curriculum involved in pursuing advanced graduate degrees or job opportunities to those unfamiliar with the process. Through a CCW lens, many transfer students would be described as entering academia with low capital. For instance, many students without family or community academic connections may be unaware that, in the United States, many STEM PhD programs provide a livable stipend and tuition for the duration of study, information that can critically alter an individual’s career plans. These seminars were structured to familiarize transfer students with this helpful information that is often not taught in their classes. One-on-one mentorship meetings were largely unstructured, though suggested topics for discussion at individual meetings were included in group discussion slides and in follow-up emails after group discussions. Additional details on these facets of the TSMP, their availabilities, and offerings are provided in Table 2.

**Table 2:** Offerings and Availability of the TSMP

Program Component	Description	Availability
Seminars	Structured mentorship and community building	All transfer students in the Departments of Chemistry and Chemical and Biomolecular Engineering
One-on-one mentorship	Unstructured, personalized mentorship and fast-tracked networking	Incoming transfer students in the Departments of Chemistry and Chemical and Biomolecular Engineering only

### Program Participation

Of the transfer students that signed up for the TSMP, all 35 juniors were matched with a graduate student mentor for the semester. Based on graduate student mentor survey responses, approximately 85% of mentees met with their mentors at least once over the course of the fall semester. Among both graduate and transfer student respondents, the median number of individual meetings per student–mentor pair over the course of the semester was three with a median absolute difference of 1 (Figure S1). Of the mentors in our program responding to the survey, nine of the 13 graduate student mentors had previous one-on-one mentorship experience, which included instances such as mentorship of an undergraduate and/or a more junior graduate student in their research lab, tutoring, and participation in a formalized mentorship program such as a mentorship program pairing incoming graduate students with current graduate students in small mentorship groups. All mentors were expected to have had experience as a teaching assistant based on a graduate student teaching requirement. Of the six total seminars hosted, both transfer student and graduate student mentor respondents reported attending a median of three seminars with a median absolute difference of zero for graduate student mentors and 1.5 for transfer students.

### Survey Design and Study Populations for Sense of Belonging Investigation

We invited participants in the TSMP program as well as transfer students not in the TSMP to engage in a survey-based investigation of sense of belonging. TSMP students, non-TSMP transfer students, and mentors were invited to participate in the survey. Surveys were approved by the university’s Institutional Review Board (IRB Protocol ID: 2021-07-14517) and distributed electronically via Qualtrics at the beginning and end of the program, corresponding to August and December of 2021 and March of 2022.

Inspiration for a survey-based assessment on sense of belonging drew from work in the Berkeley Department of Chemistry. Images used as part of this study were used as-is or with adapted captions from previously published work made available for unrestricted use under a Creative Commons Attribution 4.0 International Public License.^29^ Ten questions asking for a Likert scale-type response to cartoons paired with statements reflecting sense of belonging in a graduate program were used to assess sense of belonging of both transfer and graduate students. While the statements used had previously been validated as measuring sense of belonging in a graduate student population, they had not previously been used to measure sense of belonging in undergraduates. To probe the internal consistency of our scale when measuring sense of belonging of undergraduates, we calculated Cronbach’s α for both the beginning and ending Likert-type survey responses after inverting responses to statements corresponding with a negative sense of belonging so that a high score always indicated higher sense of belonging. The responses were found to be consistent with the exception of the phrase “I’m grateful to have a supportive social network” for both transfer and graduate student respondents, with Cronbach’s α values of 0.72 or higher for all populations (Tables S1–S4). We additionally included qualitative questions to allow respondents to further clarify feelings of sense of belonging and science identity in their own words. These questions along with a selection of responses are outlined in Table 3 and Table 4. All statistical analyses were performed in Microsoft Excel for Mac, Version 16.59.

**Table 3:** Excerpts from Student Responses to “What Does It Mean to Feel ‘At Home’ Somewhere? How Does This Feeling Apply to Your Experience in the [Departments of Chemistry and Chemical and Biomolecular Engineering]?”

Response Excerpts
“The [Department of Chemistry/Chemical and Biomolecular Engineering]... is very competitive and I constantly feel like I’m being “tested” about whether or not I’m worthy of being here and it’s generally very stressful.”
“Home is... where I feel accepted, seen, and valued... my advisor in the [Department of Chemistry/Chemical and Biomolecular Engineering] has made me feel this way and so have the students I am surrounded by.”
“As a new transfer student... I still feel very overwhelmed with the coursework. However, I do feel as though I fit in among the other transfer students in my courses/major.”
“a network of amicable and [likeminded] peers [would] make me feel much more at home...”
“I don’t really typically feel at home at the [Department of Chemistry/Chemical and Biomolecular Engineering] because I don’t really think I’m like a lot of the students here.”

**Table 4:** Select Responses to the Questions “What to You Constitutes a Scientist/Engineer? Do You Describe Yourself as a Scientist/Engineer? Why or Why Not?”^a^

Response number	What to you constitutes a scientist/engineer?	Do you describe yourself as a scientist/engineer? Why or why not?
1	“A scientist is anyone that systematically attempts to understand a process.”	“I would describe myself as a scientist as I constantly attempt to understand how things work...”
2	“...a scientist is someone who strives to discover ways to improve the world.”	“I think I’m scientist because I have a passion for using science to improve my community.”
3*	“A scientist is someone whose curiosity leads to discovery.”	“I would describe myself as a scientist.”
4	“an engineer... works to solve problems and gets paid for that work.”	“I feel like I am learning to be [an engineer], but I am not one yet.”
5	“...an engineer is someone with experience and education designing and analyzing processes.”	“I don’t... consider myself a full engineer yet because I don’t have... hands-on experience...”
6*	“An engineer is one who is... enthusiastic about exploring the unknown [via] STEM.”	“I do not feel as though I have enough knowledge to be considered an engineer.”

a Student responses 1–3 are from chemistry majors, and student responses 4–6 are from Chemical and Biomolecular Engineering majors. Asterisks (*) denote responses from students who did not participate in the TSMP.

Ten (10) TSMP undergraduates and 13 graduate student mentors completed the August survey, 12 TSMP undergraduates and 13 graduate student mentors completed the December survey, and 16 graduate student mentors completed the follow-up survey in March. This corresponds to 29% of the TSMP participants and 65–80% of the graduate student mentors. In addition, we recruited eight and seven students from the general transfer student population to participate in the initial and final surveys, respectively, as a comparison group (non-TSMP). Nine transfer students and 10 graduate students participated in both the initial and final surveys; comparisons of survey responses over time are displayed only for these students. The majority of transfer student respondents were juniors (15, 90%). Of the 14 transfer student respondents who answered the optional demographic questions, the majority were first in their immediate family to attend college (10, 71%).

### Exploration of Sense of Belonging

**Figure 3:**
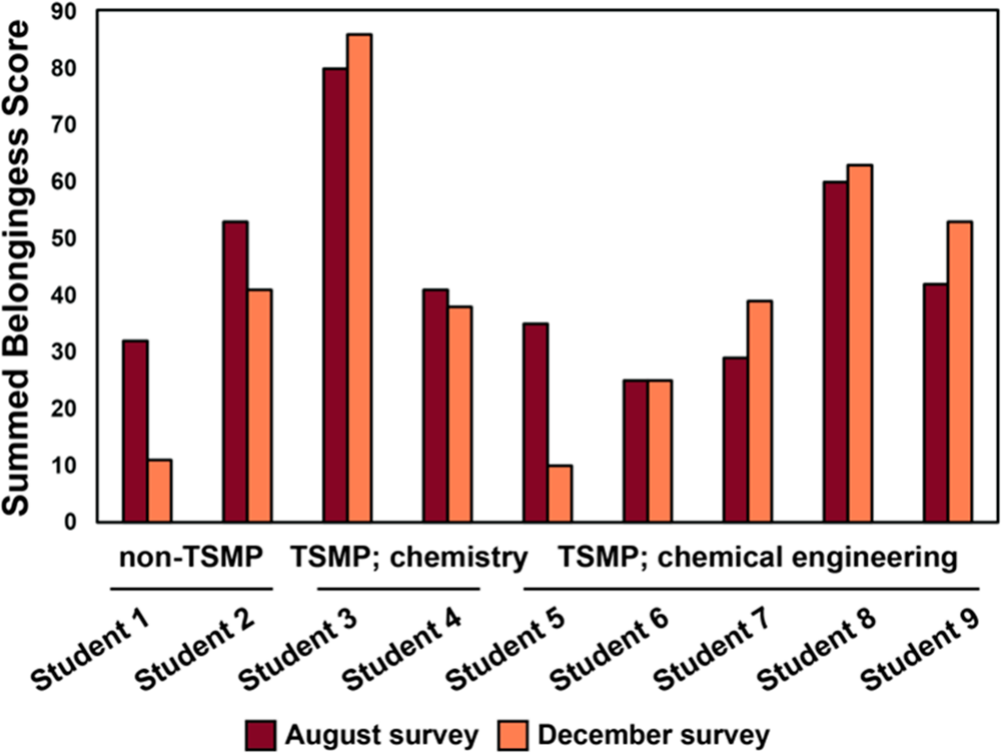
A comparison of matched student responses to the belongingness survey, sorted by participation in the program followed by major. In general, respondents that participated in the TSMP experienced improvement or consistency in their sense of belonging compared to respondents who did not participate in the TSMP. *n* = 9.

To probe the sense of belonging of survey respondents, we used a summated scale of responses to the nine internally consistent Likert-type questions. This scale added the 0–10 rankings of students’ agreement with statements related to their sense of belonging in the Departments of Chemistry and Chemical and Biomolecular Engineering. A maximum score of 90 on the summated scale correlates to the highest sense of belonging. The sense of belonging of respondents in general improved over the semester if they participated in the program (Figure 3). Students 1, 2, and 5 reported a −20% change or greater in sense of belonging by the concluding survey, with the remaining students experiencing changes from −7% to as high as +34%.

Students 1 and 2, who provided the only matched responses from transfer students that did not participate at all in the TSMP, both exhibited a marked decrease in sense of belonging from the beginning to end of their first transfer semester. A decrease in belongingness among transfer students is consistent with previous research indicating that many transfer students experience “transfer shock” during their first semester, reporting lower confidence in their institutional knowledge after the first semester begins than before they have attended the first semester at their new institution.^30^ The majority of respondents who participated in the TSMP report either little change or bettering of their sense of belonging over the course of the first semester.

While the low participant number prevented quantitative comparison of TSMP participants and transfer students who did not participate, we additionally collected qualitative answers to a question investigating belongingness in the Departments of Chemistry and Chemical and Biomolecular Engineering (Table 4). Responses draw from all populations across both the initial and final surveys, with individual responses available in the Supporting Information (Table S5). The five responses highlighted here were chosen to reflect a variety of reasons why respondents felt they did or did not belong, as well as to point out trends that were observed. Many respondents used words like “accepted”, “fit-in”, or “like-minded” to describe instances where they felt high belongingness, and some specified that these applied only within the sphere of other transfer students or their previous college. Some respondents that felt low belongingness mentioned feeling tested by their peers or that they did not belong. Interestingly, several respondents clarified that while they did not currently feel a high sense of belonging in the Departments of Chemistry and Chemical and Biomolecular Engineering, they were hopeful that they one day would.

Though this program provided a channel to improve connectivity between transfer students, graduate student mentors, and senior transfer student mentors, it did not introduce FTIC students in any manner, a group many respondents highlighted as the “in group” in their responses regarding belongingness. It follows that the TSMP would do little to affect how participants view themselves as part of this “in group”, as the program’s design does not incorporate FTIC undergraduate students.

### Exploration of Science Identity

Akin to sense of belonging, we collected responses to a qualitative short response question asking students to first define a scientist or engineer (depending on their major) and then explain whether they identified as meeting this definition themselves. Six students’ responses to these questions, separated by major, are highlighted in Table 4. All responses are available in the Supporting Information (Table S6). Chemistry-major respondents regardless of TSMP participation tended to identify themselves as scientists, while the majority of Chemical and Biomolecular Engineering-major respondents did not identify themselves as engineers.

### Rates of Research Participation

Undergraduate research participation is known to correlate with a higher sense of belonging and science identity.^31−33^ We sought to improve the science identity and sense of belonging of transfer students by increasing rates of research participation through connections made in the TMSP.

**Figure 4:**
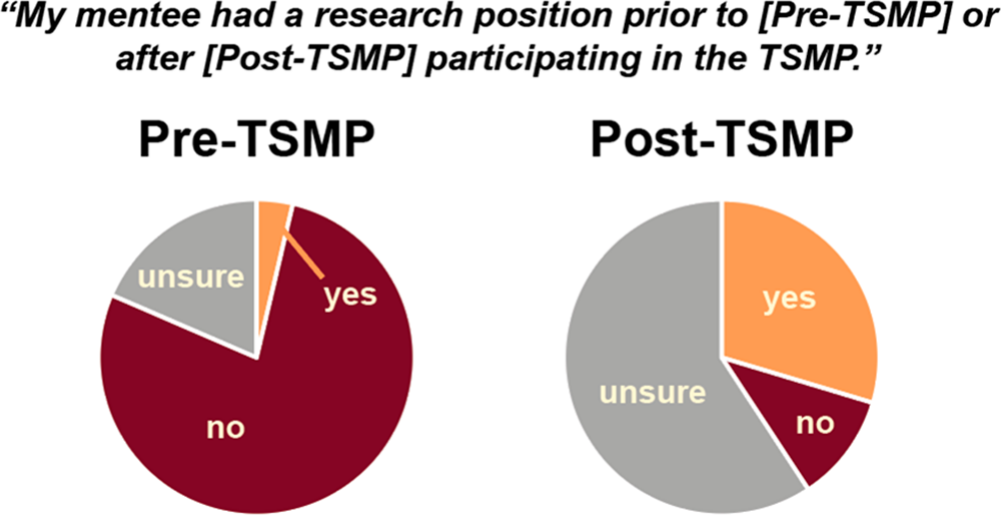
Responses of TSMP mentors to questions asking whether their mentee had a research position before or after participating in the TSMP. Data was collected three months after conclusion of the program for 27 mentees. *n* = 16 graduate student mentor respondents.

Graduate student mentors were contacted three months after the conclusion of the TSMP to comment on the status of their mentee(s) and their research participation. We received responses from 16 mentors (80% response rate), corresponding to the status of 27 mentees due to some mentors being paired with two mentees (Figure 4). While only one mentee was known to have held a research position prior to the program, seven mentees were known to have such positions at its conclusion. The number of “unsure” responses from mentors increased largely between the two time points, which is consistent with separate data collected on the number of meetings between each mentor and mentee and may indicate an attrition of mentor–mentee relationships after the conclusion of the program.

Our research additionally identified an unmet need in the department regarding Chemical and Biomolecular Engineering students and undergraduate research. Survey respondents in this population report high interest in performing undergraduate research yet consistently struggled to find these positions (Figure S2). These numbers reflect the high number of undergraduate Chemical and Biomolecular Engineering majors and low number of available research positions in the Department of Chemical and Biomolecular Engineering (Figure S3).

### Exploration of Graduate Student Experiences

Peer mentorship among graduate students has been demonstrated to academically and professionally benefit both mentored and mentoring students.^34^ We sought to further understand graduate student mentors’ experiences by administering surveys at the beginning and conclusion of the TSMP asking for short answer responses and level of agreement with cartoons assessing sense of belonging.

Graduate student mentor respondents self-reported little change in their sense of belonging between the beginning and end of the program (Figure S4). In response to a question regarding mentors’ professional development, some respondents suggested that the program had built their confidence in mentoring. Several respondents also used these questions as an opportunity to discuss how they learned about the transfer student experience or how they would like to support transfer students in pursuing research positions in the future (Tables S11 and S12). While the graduate student mentors did not provide responses indicating that they thought their future careers may benefit from their participation in the program, they did cite more knowledge of how to help transfer students in their career trajectories after being part of the TSMP.

### Limitations

The low response rate to some surveys as well as self-selection for TSMP and non-TSMP groups rather than random assignment are contributing limitations in this study. In particular, the voluntary nature of signing up for and participating in this program, rather than random assignment of students to the program, makes it difficult to disentangle whether positive benefits of participants were primarily due to involvement in the TSMP or outside factors. Improved responses across all populations would provide a more generalizable picture of changes in research participation, science identity, and belongingness. Assessment of research participation among these populations may be improved by the addition of a question surveying this experience in a survey that all undergraduate students in the Departments of Chemistry and Chemical and Biomolecular Engineering must complete prior to graduation, which will be implemented in future research.

Additional limitations include the short timeline of the study and including only a single university in the study. In particular, a large graduate student population relative to the transfer student population is likely a requirement for a near-peer mentoring program like the TSMP to effectively pair graduate students with transfer students. Because of this, similar programs are likely to be best implemented at large research institutions and would need to be adapted significantly to serve primarily undergraduate institutions or universities without a significant graduate student population. As a pilot program, this study also only followed transfer students over the course of one semester; future research will explore the persistence of any program benefits.

## Conclusions and Implications

This work introduces a framework for the implementation and evaluation of a graduate student-designed and led mentorship program at a large research institution. Near-peer mentorship and research experiences are known to have a positive effect on students’ persistence and sense of belonging in STEM,^6,10,11,15,19^ and the TSMP combines these elements to provide support for transfer students during their first semester. Recruitment of mentors and mentees was accomplished primarily via email. The key programmatic elements of the TSMP were (1) one-on-one mentorship meetings between graduate student mentors and transfer student mentees, and (2) group discussions led by graduate students and senior transfer students to provide information about academic research opportunities and an open space for asking and answering questions. Evaluation of the program adapted an existing sense of belonging scale for graduate students^29^ combined with short answer questions, which synergistically provided information about student perceptions and avenues for improvement or future research.

Graduate student mentors consistently participated in group seminars and initiated one-on-one meetings with their transfer student mentees over the course of the program. Although signing up for and subsequently participating in the program was completely optional for transfer student participants, the majority met one-on-one with their mentors multiple times throughout the fall semester. In contrast, voluntary attendance at scheduled group seminars was low. Offering these seminars as part of a formal course or providing additional incentives for attending may be necessary to heighten attendance in future iterations of the program. Additionally, formalizing knowledge about applying for and obtaining research positions and career planning through its incorporation into a course may be a way to expand the population served by this program to include FTIC students or other students without a large amount of cultural capital.

Transfer student respondents in the belongingness survey generally reported higher agreement with metrics measuring their sense of belonging by the conclusion of the program if they had participated in the TSMP. Further research is necessary to determine whether this or similar programs can serve to mitigate transfer shock or have persistent effects on transfer students’ sense of belonging at their transfer institution and department. Assessing which aspects of the program are responsible for these changes would benefit the community in the design of future programs aimed at smoothing the transition of transfer students during their first semester. Another area of interest for further study is how persistent the mentor/mentee relationship remains after the conclusion of the program and how this affects students’ future persistence and sense of belonging in STEM.

A strength of a mentorship program fitting the TSMP model is that it requires few resources and can easily be replicated at other institutions. The program was designed and directed by graduate students, with periodic faculty advising. Mentor and participant recruitment was accomplished via email and an information session during the summer before the program. While time commitment was minimal for students and mentors (approximately 10 h over the course of the semester), it provided mentorship and peer connections for nearly half of the incoming class of transfer students to the institution’s Departments of Chemistry and Chemical and Biomolecular Engineering. This type of mentorship program is expected to be most effective at institutions with a low faculty to undergraduate student ratio, where many students may struggle to find direct mentorship from faculty. Additionally, a sufficient number of graduate student mentors is required to serve as many transfer students as wish to participate in the program. However, it may be adapted to other universities by recruiting mentors from recent alumni if there is not a sufficient graduate student mentor pool. The TSMP provides a framework by which transfer students to four-year institutions, a population that often includes groups historically underrepresented in STEM, can be connected with mentors and resources to help them in their STEM educational and career pursuits.

## Supporting Information Available

Additional programmatic data, survey scales, survey responses, and survey questions (PDF, DOCX)
