# Supplementary material for: A Pilot Graduate Student-Led Near-Peer Mentorship Program for Transfer Students Provides a Supportive Network at an R1 Institution
Source: J Chem Educ. 2022 Nov 10;100(1):134–42. doi: 10.1021/acs.jchemed.2c00427 (PMC9835829; doi:10.1021/acs.jchemed.2c00427)
Supplement: Supplementary file 1 — ed2c00427_si_001.pdf [file ed2c00427_si_001.pdf]

Supporting Information

A Pilot Graduate Student-Led Near-Peer Mentorship Program for Transfer Students Provides a Supportive Network at an R1 Institution

Audrey G. Reeves[*,†,#], Amanda J. Bischoff[*, †,#,§], Brice Yates[†]; Daniel D. Brauer[†]; Anne M. Baranger[†,‡]

[†]Department of Chemistry, University of California, Berkeley, CA, USA, 94720

[#]Molecular Biophysics and Integrated Bioimaging Division, Lawrence Berkeley National Laboratories, Berkeley, CA, USA, 94720

[‡]Graduate Group in Science and Mathematics Education, University of California, Berkeley, CA, USA, 94720

*Address correspondence to these authors.

Audrey G. Reeves email: areeves@berkeley.edu

Amanda J. Bischoff email: amanda_bischoff@berkeley.edu

mentorship program, transfer students, minorities in chemistry, public outreach

The following document includes:

Figure S1: Distribution of number of individual mentorship meetings per mentee	3
Figure S2: Research position status of transfer student study participants over time	4
Figure S3. The institution's departmental demographics	5
Figure S4. Summed belongingness scores of graduate student mentors	6
Table S1: Agreement item numbering for sense of belonging scale	7
Table S2: August survey levels of agreement with sense of belonging statements on a 1-10 scale	8
Table S3: December survey levels of agreement with sense of belonging statements on a 1-10 scale	9
Table S4: Cronbach's α values for sense of belonging scale	10
Table S5: Transfer student responses to experience question 1	11
Table S6: Transfer student responses to experience question 2	12
Table S7: Transfer student responses to experience question 3	13
Table S8: Transfer student responses to experience question 4	14
Table S9: Graduate student responses to experience question 1	15
Table S10: Graduate student responses to experience question 2	16

Table S11: Graduate student responses to experience question 3 17
Table S12: Graduate student responses to experience question 4 18
Table S13: Graduate student responses to experience question 5 19
TSMP Transfer Student Pre-Program Survey (August 2021) 20
TSMP Transfer Student Post-Program Survey (December 2021) 34
TSMP Graduate Student Pre-Program Survey (August 2021) 50
TSMP Graduate Student Post-Program Survey (December 2021) 63
TSMP Graduate Student Follow-Up Survey (March 2022) 78
References 81

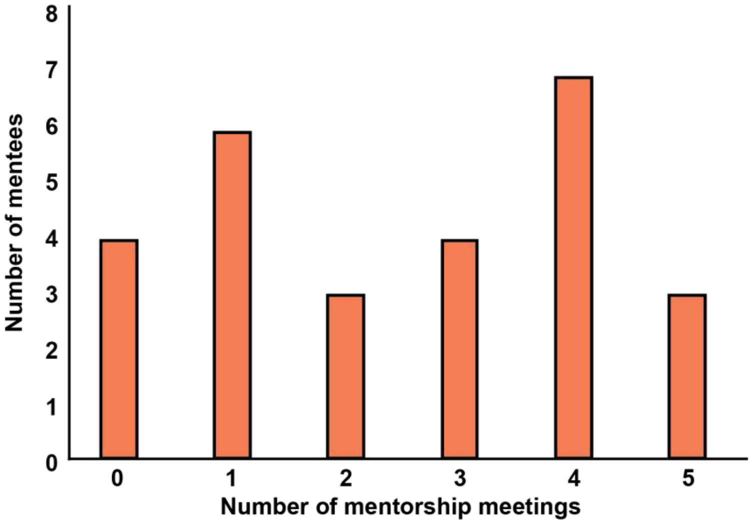

Figure S1. Distribution of number of individual mentorship meetings per mentee. Graduate student mentors were surveyed on how many mentorship meetings they had with each of their mentees, and 80% of graduate students (16 of 20) responded, corresponding to 77% (27 of 35) mentees who signed up for the program. The number of mentorship meetings exhibits a bimodal distribution with maxima at 1 and 4 meetings, suggesting that program participants settled into two primary groups; one group that engaged continuously with the program over the course of the fall semester, and a second group that did not connect with or lost interest in the program after initial meetings.

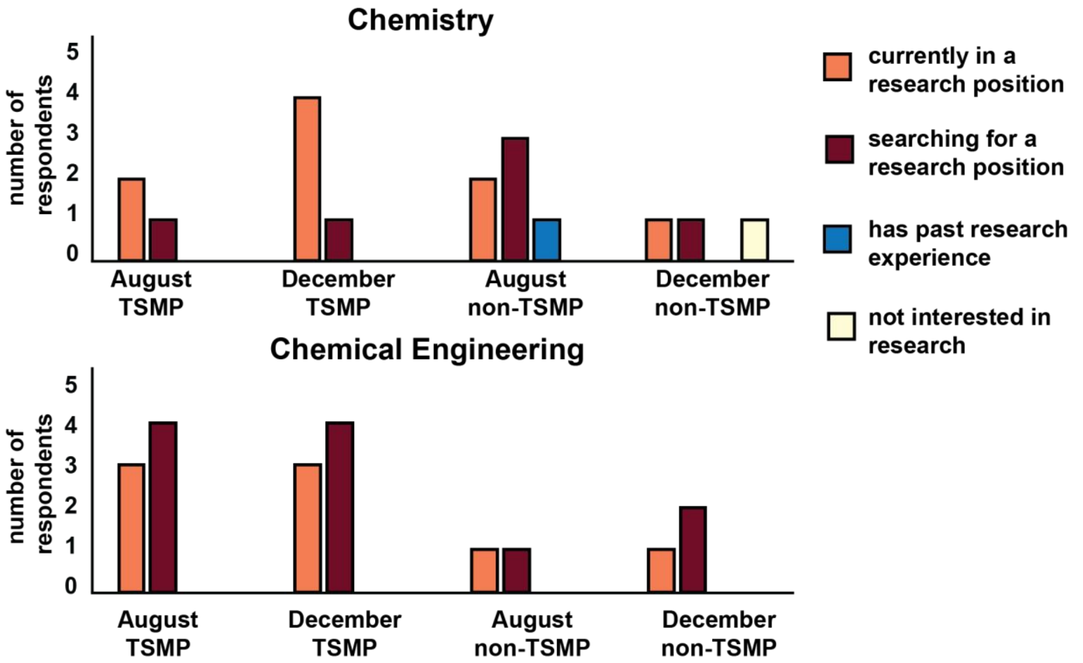

Figure S2. Research position status of transfer student study participants over time. A greater proportion of chemical engineering transfer student respondents reported searching for a research position than chemistry transfer student respondents in general, and a greater proportion of TSMP participant respondents reported obtaining research positions both during the pre-program and post-program surveys. This data includes respondents who answered both the August and December surveys in addition to respondents who only answered one of the two surveys, so August and December results are not directly comparable.

The Berkeley College of Chemistry departmental demographics (Chemistry versus Chemical and Biomolecular Engineering).

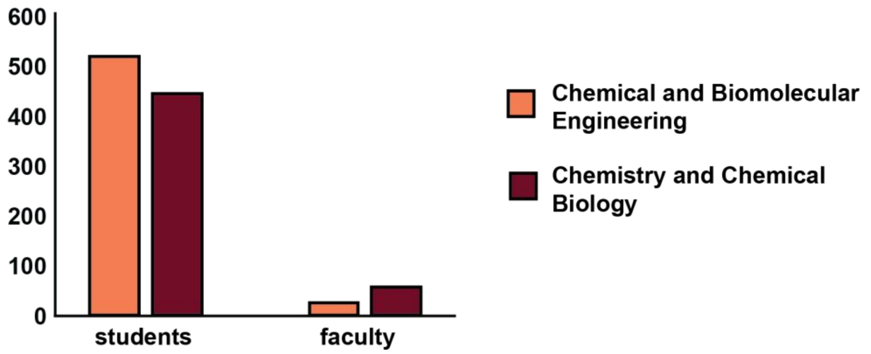

****using Spring 2021 enrollment numbers***

*****only faculty with active research groups are included***

Figure S3. The Berkeley College of Chemistry departmental demographics. The ratio of the College of Chemistry student body that is in the Department of Chemical and Biomolecular Engineering (CBE) versus Chemistry or Chemical Biology (Chem) is much higher than the ratio of faculty in CBE versus Chem. These numbers were collected from Spring 2021 enrollment numbers for students, and collected from the College of Chemistry faculty page for faculty with active research groups. Because CBE has a much lower faculty to student ratio than Chem, it is expected that there are fewer research positions available per CBE student than per Chem student.

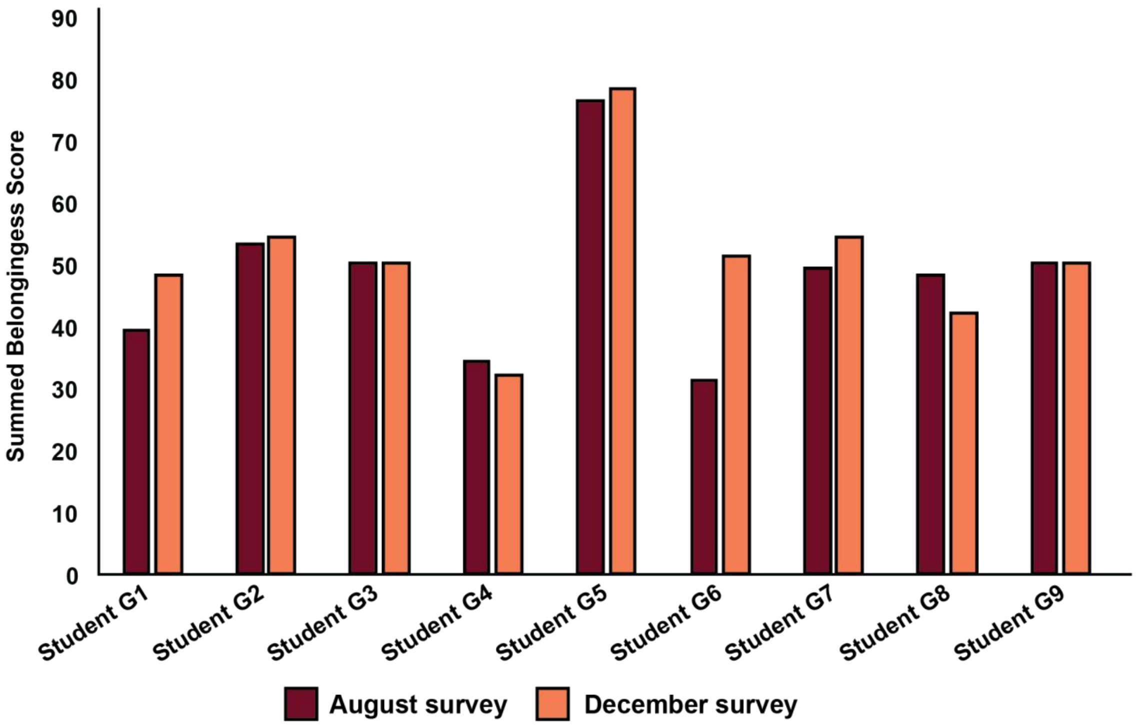

Figure S4. Summed belongingness scores of graduate student mentors. The ratings to Likert-type survey questions of graduate student mentors who completed both the August and December surveys were summed to produce an overall sense of belonging score, with the exception of the survey question "I am grateful to have a supportive social network" which was not internally consistent with the other questions. While one student (Student G6) showed a marked increase in sense of belonging score over time, most students did not display large changes in their sense of belonging score. The prefix G is used to clarify that this data displays graduate student responses.

Table S1: Agreement item numbering for sense of belonging scale		
Agreement item number	Caption for Transfer Students	Caption for graduate students
1	My classmates probably get much better grades than I do…	My classmates probably get much better grades than I do…
2	I'm definitely not smart enough to be here… (in chemistry class)	I'm definitely not smart enough to be here… (in chemistry class)
3	I wish there were more faculty I could talk to who would understand the hardships I face.	I wish there were more faculty I could talk to who would understand the hardships I face.
4	I am grateful to have a supportive social network	I am grateful to have a supportive social network
5	I feel so happy and accepted here!	I feel so happy and accepted here!
6	Other students are more productive and scientifically successfully than I am	Other grad students are more productive and scientifically successfully than I am
7	I feel like my audience sees me as a serious scholar!	I feel like my audience sees me as a serious scholar!
8	I feel like an outsider. (next to chemistry building)	I feel like an outsider. (next to chemistry building)
9	My instructor values my ideas and respects me.	That was a productive meeting… I'm so glad my advisor values my ideas!
10	I am an independent, confident scientist!	I am an independent, confident scientist!

Table S2: August survey levels of agreement with sense of belonging statements on a 1-10 scale										
Agreement Item	1	2	3	4	5	6	7	8	9	10
Student T1*	6	6	6	3	3	6	3	6	3	3
Student T2	6	6	5	4	6	4	6	3	8	7
Student T3	1	1	1	8	10	3	8	0	8	10
Student T4	8	7	8	7	7	6	5	5	7	6
Student T5	7	7	8	7	6	8	6	8	8	3
Student T6	10	10	10	10	5	10	0	5	10	5
Student T7	8	7	8	7	4	7	5	8	5	3
Student T8	8	7	3	9	9	7	9	0	8	9
Student T9	8	8	5	9	8	9	6	7	8	7
Student T10	10	10	10	10	10	10	10	10	10	10
Student T11	5	0	1	10	5	5	5	0	10	10
Student T12	0	0	0	8	9	0	9	2	8	9
Student T13	10	10	8	7	6	8	4	6	5	4
Student T14	10	10	7	3	3	8	3	9	5	4
Student T15	3	5	4	7	6	4	-	3	7	6
Student T16	6	6	3	10	4	6	7	4	6	8
Student T17	9	10	4	7	10	10	7	6	7	3
Student T18	8	8	5	6	7	9	4	5	5	3
Student G1**	7	7	4	5	5	7	4	5	5	5
Student G2	1	4	4	8	8	7	5	4	7	3
Student G3	7	5	5	8	8	8	6	3	7	7
Student G4	7	7	6	6	5	10	3	7	7	6
Student G5	0	0	3	10	10	1	4	0	9	7
Student G6	5	7	7	4	2	7	4	7	4	4
Student G7	7	7	7	6	7	-	8	5	10	7
Student G8	3	6	2	8	7	10	7	5	4	7
Student G9	7	7	3	8	6	7	7	3	7	5
Student G10	3	2	6	6	7	8	5	5	6	6
Student G11	6	7	5	8	7	9	4	5	7	6
Student G12	5	5	2	7	3	7	4	2	3	6
Student G13	2	1	3	7	7	6	5	1	5	5

* "T" prefix stands for transfer student

** "G" prefix stands for graduate student

Table S3. December survey levels of agreement with sense of belonging statements on a 1-10 scale										
Agreement Item	1	2	3	4	5	6	7	8	9	10
Student T1*	9	9	9	5	1	8	2	10	2	1
Student T2	6	7	7	5	6	7	6	4	5	5
Student T3	2	0	0	10	10	2	10	0	10	10
Student T4	8	8	8	6	6	6	5	7	7	7
Student T5	10	10	10	8	4	9	0	9	4	0
Student T6	10	10	10	5	5	10	2	7	10	5
Student T7	7	0	8	3	2	8	6	7	7	4
Student T8	8	6	8	9	9	2	10	0	8	10
Student T9	7	5	3	8	7	6	8	5	7	7
Student T20	4	2	0	7	8	3	5	1	7	6
Student T21	4	3	3	5	7	4	5	3	10	6
Student T22	3	3	3	3	3	3	3	3	5	5
Student T23	10	7	8	7	8	10	3	9	9	7
Student T24	4	4	4	8	8	4	-	-	8	8
Student T25	3	0	10	0	0	0	10	5	2	10
Student T26	9	10	10	3	2	9	2	9	9	6
Student G1**	5	5	2	8	5	10	6	3	7	5
Student G2	0	5	7	8	8	7	7	2	7	3
Student G3	7	7	3	7	7	8	6	3	8	7
Student G4	8	9	6	9	7	9	2	7	8	4
Student G5	1	0	3	5	9	3	7	1	10	10
Student G6	0	6	6	3	4	2	6	7	6	6
Student G7	8	8	8	7	7	4	8	8	10	6
Student G8	2	6	2	8	8	10	7	3	6	6
Student G9	6	6	6	8	5	6	6	5	6	4
Student G10	3	2	7	7	7	9	4	3	8	5
Student G14	6	8	4	9	9	10	8	6	7	6
Student G15	3	5	2	8	8	6	6	3	8	7

* "T" prefix stands for transfer student
** "G" prefix stands for graduate student

Table S4. Cronbach's α values for sense of belonging scale		
Agreement items	Population	Cronbach's α
1-10	Graduate students August survey	0.86
1-10	Graduate students December survey	0.62
1-10	Transfer students August survey	0.89
1-10	Transfer students December survey	0.88
1-3, 5-10	Graduate students August survey	0.83
1-3, 5-10	Graduate students December survey	0.72
1-3, 5-10	Transfer students August survey	0.90
1-3, 5-10	Transfer students December survey	0.90

Table S5. Transfer student responses to experience question 1: "Think about what it means to you to feel "at home" some - where. How does this feeling apply to your experience in the [Departments of Chemistry and Chemical and Biomolecular Engi - neering]?"		
August survey		
Responses	***TSMP***	***Major***
"excellent"	no	Chem
"I feel like I can comfortable express the way I feel and act when involved with others in the [Department]."	no	Chem
"I wouldn't quite say I feel at home at [this instiution] yet, but I am definitely getting there. Once I can establish a network of amicable and like minded peers I believe I will feel much more at home [here]."	no	Chem
"When I was at [my community college], I had my own community over there. I want to have the same kind of community, but even better at the [Department]."	no	Chem
"There is a good amount of support here, with the different programs such as TSMP itself or CAMP."	no	Chem
"To me, feeling "at home" somewhere means I am comfortable enough to say what's on my mind with less of a filter than I would normally have in something like an academic setting. The [departments of chemistry and chemical engineering at this institution] is very competitive and I constantly feel like I'm being "tested" about whether or not I am worthy of being here and it's generally very stressful."	no	ChemE
"I dont think that my experience in the [Department] is quite at "home" level. It could definitely get there! It's just not been enough time. Through spending more time on campus, making more friends, having set study spots, and getting to know faculty more, I think campus could feel like home to me."	no	ChemE
"To feel "at home" somewhere, I need to be comfortable with my surroundings. Applying to my experience in the [Department], it has been a bit difficult adjusting, but I value making use of resources given such as the [Department] Library, as well as the Peer Tutoring Center."	yes	ChemE
"As a new transfer and less than a month into my first semester, I still feel very overwhelmed with the coursework. However, I do feel as though I fit among the other transfers in my courses/major."	yes	ChemE
"I'm not there yet. I would probably feel at home after struggling through classes with other people who I become friends with."	yes	ChemE
"To me, feeling at homes comes with a sense of safety and protection. Home is a place where I feel accepted, seen, and valued. I feel like, thus far, my advisor in the [Department] has made me feel this way and so have the students I am surrounded by."	yes	ChemE
December survey		
Responses	***TSMP***	***Major***
"Feeling "at home" at [this institution] means to adjust to how to succeed in class and to feel like a part of the community of the college."	no	Chem
"Have a support network, meaningful activities to do and people to do them with. No need to feel I have to act a certain way."	no	ChemE
"The staff has been very friendly. I feel like I can go up to anyone and ask for help."	no	ChemE
"I feel like a valued member of my classes and the lab I am performing research in."	yes	Chem
"I don't really typically feel at home at the [Department] because I don't really think I'm like a lot of the students here. But honestly the majority of the students here are insecure and have toxic competitive mindsets."	yes	Chem
"There are many supportive students if you make the effort to maintain good [relationships]."	yes	Chem
"I feel like [the Department] isnt home yet. I havent made friends yet. the ones I thought I made would just take homework answers from me and use me. I asked if I can join their study group and they said yes but never invited me. they talked about how they met up ever Wednesday and Thursday at the end of the semester but never asked me to join. they would just ask me for answers and never help me if I was stuck on an answer."	yes	ChemE
"Having faculty and classmate/friends to work with homework and other problems on. I have sort of experience it but it's sort of halfway."	yes	ChemE
"I haven't felt at home yet, I think it is until we find our social circle both in friends and mentors that we start feeling at home and supported but I haven't found any of that yet"	yes	ChemE
"Unsure- to me it's just school. I go and do work then go home."	yes	ChemE
"My first semester was tough which really impacted the way I view myself [at the institution]. Unfortunately, I did not feel so confident when beginning the semester but I hope feel more "at home" next semester!"	yes	ChemE
"Feeling at home to me means a place where I can feel comfortable to be myself. To be accepted for who am and respected for the things I stand for."	yes	ChemE

Table S6. Transfer student responses to experience question 2: "What to you constitutes a scientist? Do you describe yourself as a scientist? Why or why not? (For engineering students, please answer these questions for "engineer" rather than "scientist")."		
August survey		
Responses	***TSMP***	***Major***
"patient, creative"	no	Chem
"A true scientist is someone that is driven by curiosity. Instead of doing what most people do and watch movies for fun, scientists spend a lot of time thinking and using the laws of science to better understand why something is a certain way. "	no	Chem
"What defines a scientist is how they think on a daily basis. A scientist would often use the knowledge they acquire from school and apply it to everything around them (even if they aren't in the lab or in a classroom)."	no	Chem
"A scientist is anyone that systematically attempts to understand a process. I would describe myself as a scientist as I constantly attempt to understand how things work in my daily life. eg. how is coffee brewed?"	no	Chem
"I think I can describe myself as a scientist. Whenever I get approached with something, I will always have questions about it, even if I do not ask about it in person, and will do my best to look up on that topic for answers later."	no	Chem
"For me, someone who is an engineer is someone with experience and education designing and analyzing processes and applying the science of a subject to the real world on a large scale. I don't really consider myself a full engineer yet because I don't have the same hands-on experience as someone I would consider a full engineer would."	no	ChemE
"I think an engineer is someone with ideas and is able to execute such ideas. I only consider myself an engineer when I am working an internship that I am hands on with or even writing code. Currently, since i am not working in either of those circumstances, i dont consider myself an engineer, maybe a student-engineer? Unsure"	no	ChemE
"I think anyone can be an engineer, but I don't think engineering is for everyone. It's for the student who wants to be challenged and is willing and able to take on that challenge. I'm not sure if I would feel confident enough to describe myself as an engineer yet. I don't feel confident enough in the, what feels like, small amount of knowledge I have regarding the subject. "	yes	ChemE
"I consider myself an Engineer as I work to understand concepts and look for solutions in places that may not be the most evident. I am proud to be studying as a minority in the Engineering field, from all aspects of my background. "	yes	ChemE
"An engineer is one who is curious, passionate, and enthusiastic about exploring the unknown through the use of STEM. I do not feel as though I have enough knowledge to be considered an engineer however I do aspire to be one or try my best to be one."	yes	ChemE
"Engineers are problem solvers who utilize math and other knowledge respective of their particular fields. I don't think I'm that much of an engineer since I'm not particularly interested in solving problems more than wanting to build things that I find interesting or are just new ways of doing things."	yes	ChemE
December survey		
Responses	***TSMP***	***Major***
"I think a scientist is someone who strives to discover ways to improve the world. I think I'm a scientist because I have a passion for using science to improve my community."	no	Chem
"An engineer is someone who can break a problem down, understand the needs of the problem, and come up with ways to meet those needs as simply and as efficiently as possible. I would like to be there one day, but currently, I do not feel I have enough knowledge to break down many of the problems."	no	ChemE
"I feeling I am learning to be one, but I am not one yet. I think an engineer is someone who works to solve problems and gets paid for that work."	no	ChemE
"I would describe myself as a scientist. A scientist is someone whose curiosity leads to discovery"	yes	Chem
"Someone who utilizes math and various science principles to develop solutions to various problems."	yes	ChemE
"After taking my first chemical engineering course I am more confident in saying that I am an engineering in the making. I understand what engineers do, and how engineering relates to everything I have learned before"	yes	ChemE
"I do because i am constantly learning and constantly curious of my surroundings."	yes	ChemE
"not sure"	yes	ChemE
"I believe an engineer is driven, curious, enthusiastic, and passionate about STEM. I believe I am an engineer in training, however I would have liked to have a stronger STEM background."	yes	ChemE
"I describe myself as an engineer because I have always been good at analyzing and interested in innovative thinking and having an understanding of how things work. I believe I can develop on this skill and interest, and use them to solve problems and improve the way of life of others. "	yes	ChemE

Table S7. Transfer student responses to experience question 3: "Which TSMP seminar(s) did you like best? What about the seminars did you find useful?"		
December survey		
Responses	***TSMP***	***Major***
"How to get involved with research. Had many good tips. Also liked the funding one."	yes	Chem
"2 [Applying for research positions] and 3 [Introduction to research groups]. There were a lot of helpful resources for finding research and how to write an email to a PI for research opportunities."	yes	ChemE
"I liked the more industry focused ones"	yes	ChemE
"The applying for Research Seminar"	yes	ChemE

Table S8. Transfer student responses to experience question 4: "Did you feel that your TSMP graduate student mentor was well matched to you and your research interests? Please describe why or why not."		
December survey		
Responses	***TSMP***	***Major***
"Yes, she was currently working in a field I was interested in. "	yes	Chem
"Yes. They helped me improve my resume to send to professors and grad students when applying to research."	yes	Chem
"My graduate student mentor is in a different field of study but his advice and guidance with applying for research was still helpful in planning my application."	yes	ChemE
"Somewhat, she was a chemistry major "	yes	ChemE
"Not really, but i learned about life after grad from them."	yes	ChemE
"Sort of. She was pretty helpful in looking for research opportunities but there was just a general disconnect because I am in chemical engineering and she was in chemistry."	yes	ChemE
"Yes, my mentor helped as much as possible. She advised me on how to get research positions and offered me resources."	yes	ChemE
"they are great but they are not in the same major(chemE)"	yes	ChemE
"Yes because it was in the [Departments of Chemistry and Chemical and Biomolecular Engineering], but I am interested in exploring chemical engineering and she didn't have a lot of input about that since her expertise is chemistry. I still valued her advice and we talked about other things"	yes	ChemE

Table S9. Graduate student responses to experience question 1: “Think about what it means to you to feel "at home" some - where. How does this feeling apply to your experience in the [Departments of Chemistry and Chemical and Biomolecular Engi - neering]?”
August survey
Responses
“This feeling comes mostly from a sense of community, and I've found that among my peers.”
“I feel comfortable being myself in my group and like I'm welcomed here and I think those two things are what make you feel at home.”
“I don't expect to feel "at home" in a job. That said, I have enjoyed working with many of the people I've met at [this institution] and count myself very fortunate to work with some incredibly understanding and supportive colleagues.”
“I feel at home when I'm surrounded by a lot of people who I am comfortable both asking questions about work/science/school but can also talk about less "serious" stuff with them and joke around. This is very much the case for me [here].”
“Feeling comfortable and at ease (not on edge/ anxious) and feeling accepted by those around you. Feeling like you belong here. ”
“[The Department] has certain dominant cultures that I struggle to completely feel engaged in. I think a lot of that comes from being an international student, as I notice that many international students also struggle to feel part of the community. For example, I avoided [alco-hol-based social hours] my first year since I don't drink. Another example is visiting weekends everyone brags about the schools they got admitted into and where they did their undergraduate degrees. As an international student, admission to US schools is much harder, and we didn't come from schools Americans know about, making the conversation not very relevant or interesting.”
“For me, feeling at home involves being surrounded by people I can rely on to support me through both hardships and good times, and gives me a sense of comfort and belonging (and for me to support them). Being at home also involves being close to family wheth er that's chosen family or by relations and involves living in a place that I have a special connection with (somewhere I grew up, or hav e fond mem-ories of, etc.). While I feel a sense of belonging in the [Department] I would not describe it as being "at home", it is my work place and while I have many good friends in the [Department] who do support me, I still consider it a professional work environment.”
“I think feeling at home involves a lot of different things. One thing is definitely feeling like you're a successful scientist within the depart - ment that contributes to your field. More importantly though, is the social aspect of feeling at home. I think overall the depa rtment could have more social events and organizations to make people feel more at home here.”
“To feel at home for me is to be surrounded by people who know me, care about me, and value me. It took me about a year , but I do feel mostly at home in the [departments of chemistry and chemical engineering] after building relationships with my peers and collea gues.”
“I feel part of a community of scientists, and being part of a community that respects me is part of feeling at home. Feeling v alued for my contributions, but also being cared about as a person and not just what is valuable about me.”
“Simply put, home is a place that I have support from loving friends. The physical location doesn't matter that much, as long as it doesn't pose any serious threat of physical harm.”
December survey
Responses
“I feel like I have friends here and people I can just kind of chill with. Like home is somewhere you don't have to pretend to be someone and I can (usually) do that here.”
“While there are people that I really enjoy being around in my lab and in the department, I'm not sure that I feel "at home" in the [Depart - ment]. I also don't know that that feeling should be my goal in a professional environment, though I believe it an admirable goal for the [De-partment] to uphold for its students, provided that home is an upstanding one. ”
“Having a strong support system in the department and a strong social network”
“I feel included and like I know "the rules" here, and how the [Department] works, like I'm part of the community. But not entirely settled -- I feel part of but not deeply integrated into the community here.”
“I'm not entirely sure but probably has something to do with being familiar with a place in terms of people, physical space, and something more abstract than that. I would say that I have not really felt at home within the [departments of chemistry and chemical engineering], but I have within my lab. I will say that participating in the TSMP made me feel more at home within the [Department] than I did before.”
“I would say this feeling applies to my experience in the [Department], despite my lack of confidence in my ability to succeed.”
“I feel "at home" in the [Department] in that I feel welcome and included, particularly within my research group”

**_Table S10. Graduate student responses to experience question 2: "What to you constitutes a scientist? Do you describe your - self as a scientist? Why or why not?"_**
**_August survey_**
**_Responses_**
"A scientist is someone that uses the scientific method to generate knowledge. I consider myself to be a scientist because I actively do this in my research. "
"A scientist asks questions and then seeks to answer them in a methodical way . I do see myself as a scientist"
"A scientist spends time thinking about and working on scientific problems. My work focuses on solving scientific problems, which I believe qualifies me to the title of scientist. "
"Someone conducting research to answer a scientific question that will further our understanding of the world. Yes, I describe myself as a scientist because this is what I'm actively doing with my graduate work."
"A person that is conducting research (or learning to conduct research) that may contribute to overall scientific progress. I fee l I fit that bill."
"I think someone who is funded by some entity to perform experiments that answer a particular question in a field. I guess can be poorly interpreted by someone who gets paid by shady alt-right/evangelical/etc. groups to do this poorly and fudge data or something m alicious, but in my eyes those people are still technically "scientists", just not "good people". I do describe myself as a scientist bec ause I feel like that is what I'm getting paid to do as a [graduate student researcher]."
"The ability to conduct very evidence-based experiments to reach the sought-after non-ambiguous truth as well as the ability to critique other science independently. I am slowly becoming a scientist as I am nearing the end of my graduate school as I am learning these skills as I go. Many people graduate not knowing how to think independently which I think is [problematic]."
"A scientist is someone who works in any kind of STEM job, comes up with hypotheses and/or tests them via experiments. I would de scribe myself as a scientist as I spend all my time doing this as a grad student."
"A scientist is someone who does (or knows) science professionally . Since my income comes from working in a lab (as opposed to under - grad) I consider myself a scientist."
"A scientist is a person who is thinking critically about problems related to science and working to solving them. As a graduate student, I believe I am a scientist and working towards being a better scientist!"
**_December survey_**
**_Responses_**
"I guess? A scientist is someone who does science and since I get paid to do science I am a scientist. I usually call myself a chemist instead but I suppose yes, I am a scientist."
"I am a frustrated scientist, but a scientist nonetheless. I don't think I'm particularly good at my job, especially the troubleshooting aspect of it, but I do it daily and try to make the best of the most interesting results of my work."
"Curiousity. Yes."
"Yes, I do describe myself as a scientist, because I'm curious about the world and do research to find answers to my questions. I used to consider myself more of a "student" than a scientist, but that has changed recently"
"I think a scientist is someone that applies the scientific method to things, which is usually in a formal setting like a lab, but I think that the scientific method can be applied to many aspects of life. I do consider myself a scientist both in occupation and in other things."
"Someone who conducts experiments to answer a question of scientific relevance. I describe myself as a scientist because this is what I have aimed to do since I started studying science."
"In a general sense, I think a scientist is anyone who is curious about the world and tries to understand it more, in whatever way. Yes, I describe myself as a scientist, because my work involves trying to explain something (very specific) about the world."
"I'd probably consider just about anyone that asks critical questions about the world around them and does something to try to answer those questions. I'd consider myself a scientist"

Table S11. Graduate student responses to experience question 3: "Which TSMP seminar did you find the most useful and why?"
December survey
Responses
"career paths--probably the most neglected part of my education until late undergraduate studies. I had a very one-track mind a nd wish I had explored more options earlier"
"tbh I didn't really find them useful"
"Applying to research positions. This is the biggest hurdle for a lot of the undergraduates."
"How to apply to research positions"
"Intro to Research Groups"
"Navigating classes in the [Departments of Chemistry and Chemical and Biomolecular Engineering], because I personally did not p reviously know much about the different options and structure of undergraduate programs here"
"Getting into a research lab"
"Finding a research group- helpful as a mentor for giving advice to my mentee"
"I only saw the full seminar for the one on finding research groups at [the institution], but it definitely seemed helpful for students. My mentee was a bit unaware of how to look into research positions on campus before that."

Table S12. Graduate student responses to experience question 4: “What has the impact of the TSMP been on your professional development?”
**December survey**
Responses
“It's been a really good experience to learn more about the transfer student experience at [the institution], and adapting advi ce and mentorship to different student's needs. The group discussions at the TSMP meetings have been particularly valuable.”
“none? I met some nice people though!”
“It was helpful to discuss research with undergrads and get their perspectives”
“It's helped me understand what goes into a mentorship role,”
“minimal, to be honest. I was unable to attend most sessions in person but would have liked to form better ties with fellow men tors/mentees”
“It was fun to chat with my mentee about his interests, since they were much more engineering focused and he taught me about so me of the engineering clubs/projects on campus that I hadn't heard of before”
“Conversations with my mentees taught me more about the issues transfer students can face in joining research groups, forming r elationships with peers/instructors, and navigating course requirements. This has motivated me to consider transfer students in particular if I end up recruiting or choosing another undergrad to work with on my research, and I plan to encourage my peers to do the same.”

Table S13. Graduate student responses to experience question 5: "Did you feel that you and your transfer student mentee were well matched? Please outline why or why not."
December survey
Responses
"My first mentee did not respond to any of my emails. My second mentee stopped responding after our first meeting. So maybe we were not well matched in my mentees' minds. The second mentee was a Chem Eng student with different research interests and career goals."
"I did! As a transfer student myself during undergrad I found the pain of the 'first semester' very relatable."
"Yes, one of them had similar interests and the other one was interested in med school which I knew about that whole application process"
"Yes, one of them in particular was interested in the same field as me and had previous experience in it. "
"I didn't feel particularly well-matched to my mentee because she is a chemical engineering major, and the job path for ChemE vs chemistry tends to be much"
"Kind of! I got along well with both of my mentees, but they were both studying chemical engineering and I'm not as well-inform ed about the specifics of chemical engineering courses, research groups, or internships. This meant that I didn't know the answers to some of their questions and was only able to give them more general answers."
"Yes, for the most part. We got along fairly well, and I felt that I was able to point her in the direction of some resources that appealed to her interest in energy specifically, and we were also able to discuss biotech options etc. However, she was in chemical engineering, and did have some questions that were more engineering-specific that I couldn't particularly help her with."
"Yes, I think me and my transfer student mentee were well matched. We have somewhat similar research interests and personalities."
"Unfortunately not applicable. The first mentee I was paired with never responded after 3 attempts to contact them. Later I was given a second mentee that seemed enthusiastic but when we tried to meet they canceled and then asked to have my social media contacts and didn't want to email and I thought that was a little odd"
"Yes and no- one of my mentees was a good match and I felt like I could talk to him about classes and research groups in a way th at he found helpful; my other mentee was an engineering major and I was very unfamiliar with his areas of interest"
"Not necessarily, since the student was a chemE major and we had relatively different interests both in research and outside of school. But it wasn't an issue at all really, I still think it went well and was (hopefully) quite helpful for the student."

TSMP Transfer Student Pre-Program Survey (August 2021)

All images used in this survey were reproduced under CC BY International 4.0 License, from ref 1. 2020, *PLOS ONE*.

1. What year are you?

- ○ Sophomore
- ○ Junior
- ○ Senior
- ○ Other ______________________________

2. What is your major?

____

3. Have you ever been involved as an active member of the Transfer Student Mentorship Program (TSMP)?

- ○ Yes
- ○ No
- ○ Other ______________________________

4. If you were involved in the TSMP, which year did you first join?

- ○ 2020
- ○ 2021

5. Please describe how you would go about applying for an undergraduate research position in a campus lab (please answer even if you already have a research position).

____

____

____

____

____

6. Please choose the option below that best describes your research experience.

○ I am working as an undergraduate researcher in a campus lab

○ I am working as an undergraduate researcher in an LBNL lab

○ I am performing research through an established undergraduate research program (i.e. BLUR, URAP). Please include the program below:

____

○ I am or have performed research through an established summer research program (i.e. Amgen scholars, SURF, REU). Please include the program below:

____

○ I am interested in performing research but have not found a position yet.

○ I have performed research in the past but am not currently in a research position

○ I am not interested in performing research

○ Other ______________________________

7. How strongly do you identify with the character on the right in the cartoon?

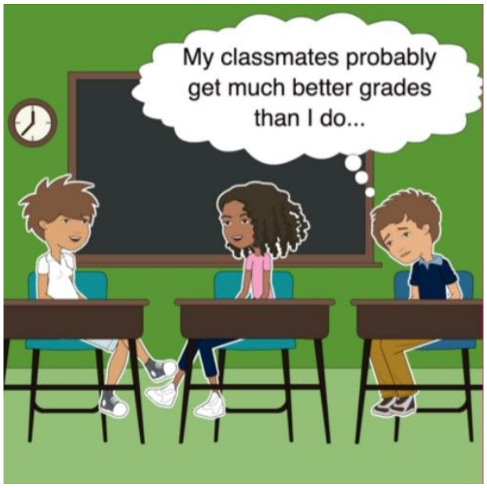

- ◯ 0 – Do not identify
- ◯ 1
- ◯ 2
- ◯ 3
- ◯ 4
- ◯ 5
- ◯ 6
- ◯ 7
- ◯ 8
- ◯ 9
- ◯ 10 - Very strongly identify

8.

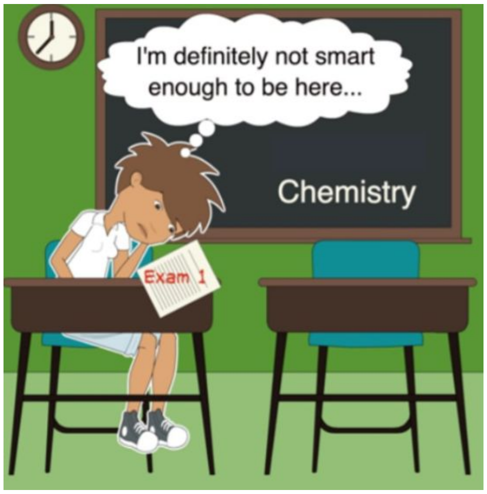

- ○ 0 – Do not identify
- ○ 1
- ○ 2
- ○ 3
- ○ 4
- ○ 5
- ○ 6
- ○ 7
- ○ 8
- ○ 9
- ○ 10 - Very strongly identify

9.

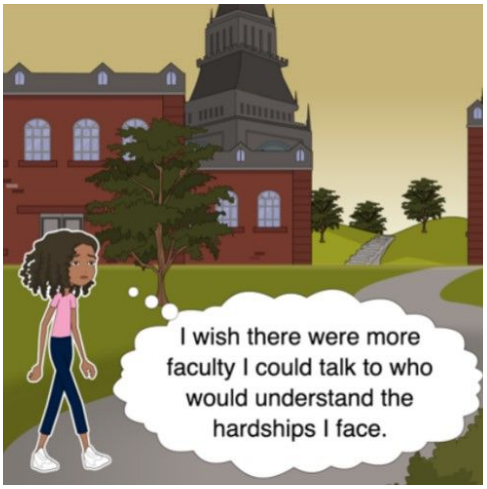

- 0 – Do not identify
- 1
- 2
- 3
- 4
- 5
- 6
- 7
- 8
- 9
- 10 - Very strongly identify

10. How strongly do you identify with the character on the left of the cartoon?

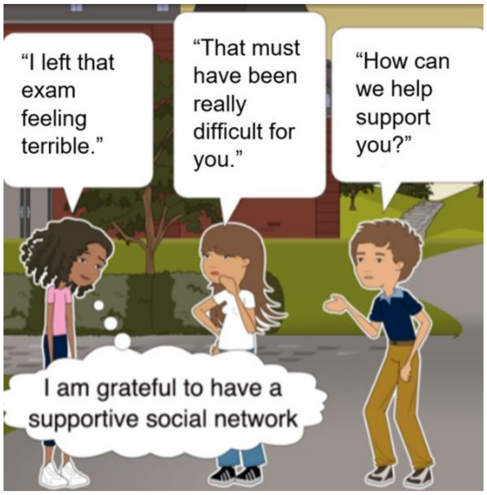

- ◯ 0 – Do not identify
- ◯ 1
- ◯ 2
- ◯ 3
- ◯ 4
- ◯ 5
- ◯ 6
- ◯ 7
- ◯ 8
- ◯ 9
- ◯ 10 - Very strongly identify

11.

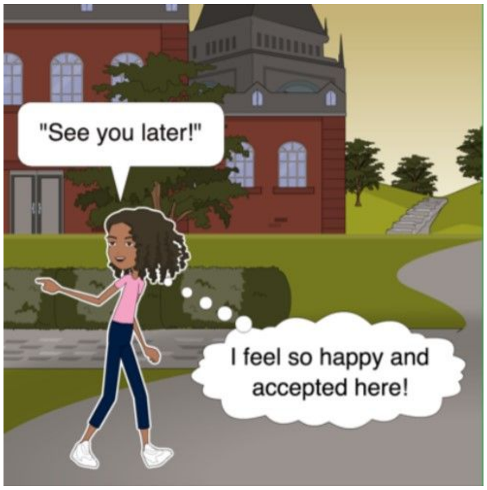

- ◯ 0 – Do not identify
- ◯ 1
- ◯ 2
- ◯ 3
- ◯ 4
- ◯ 5
- ◯ 6
- ◯ 7
- ◯ 8
- ◯ 9
- ◯ 10 - Very strongly identify

12.

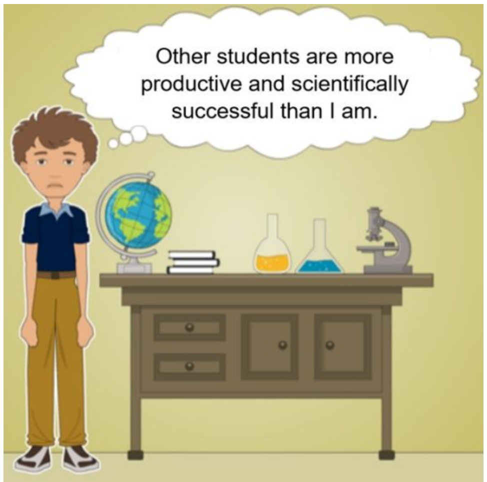

- ○ 0 – Do not identify
- ○ 1
- ○ 2
- ○ 3
- ○ 4
- ○ 5
- ○ 6
- ○ 7
- ○ 8
- ○ 9
- ○ 10 - Very strongly identify

13.

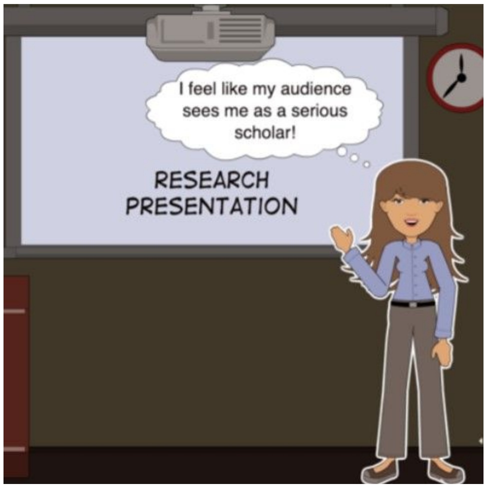

- 0 – Do not identify
- 1
- 2
- 3
- 4
- 5
- 6
- 7
- 8
- 9
- 10 - Very strongly identify

14.

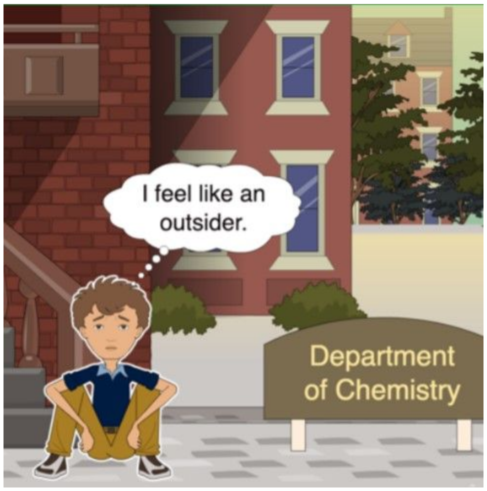

- 0 – Do not identify
- 1
- 2
- 3
- 4
- 5
- 6
- 7
- 8
- 9
- 10 - Very strongly identify

15.

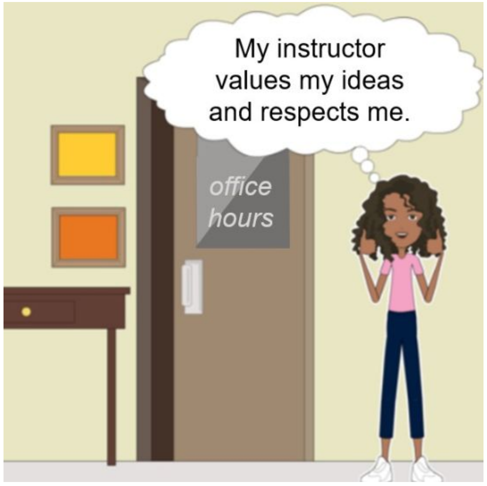

- 0 – Do not identify
- 1
- 2
- 3
- 4
- 5
- 6
- 7
- 8
- 9
- 10 - Very strongly identify

16.

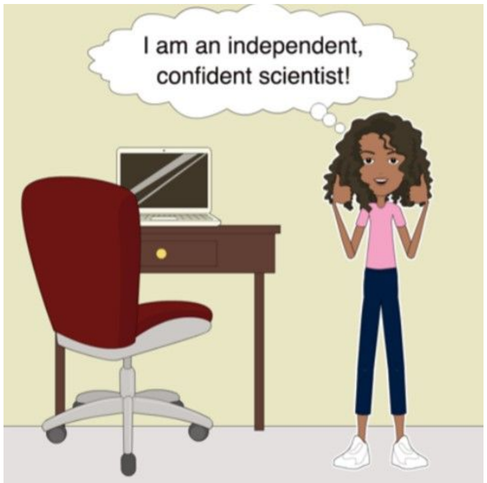

- 0 – Do not identify
- 1
- 2
- 3
- 4
- 5
- 6
- 7
- 8
- 9
- 10 - Very strongly identify

17. Think about what it means to you to feel "at home" somewhere. How does this feeling apply to your experience in the [Departments of Chemistry and Chemical and Biomolecular Engineering at this institution]?

18. What to you constitutes a scientist? Do you describe yourself as a scientist? Why or why not? (For engineering students, please answer these questions for "engineer" rather than "scientist").

Your data will be kept private and only accessible by the study directors via password-protected computers. Please keep in mind that while your data is kept as secure and private as possible, no guarantees can be made against the low risk of an inadvertent breach in confidentiality. For this reason, if there is any risk that a breach of your responses would lead to any damage or discomfort we encourage you to please leave the question blank.

19. (Optional) Please state the gender you best identify with.

____

20. (Optional) Please state the sexuality you best identify with.

____

21. (Optional) Please state the race/ethnicity you best identify with.

____

22. (Optional) Are you the first in your immediate family to attend college?

○ Yes

○ No

TSMP Transfer Student Post-Program Survey (December 2021)

All images used in this survey were reproduced under CC BY International 4.0 License, from ref 1. 2020, *PLOS ONE*.

1. What year are you?

- ○ Sophomore
- ○ Junior
- ○ Senior
- ○ Other ____________________

---

2. Have you ever been involved as an active member of the Transfer Student Mentorship Program (TSMP)?

- ○ Yes
- ○ No
- ○ Other ____________________

---

3. If you were involved in the TSMP, which year did you first join?

- ○ 2020
- ○ 2021

---

4. Please describe how you would go about applying for an undergraduate research position in a campus lab (please answer even if you already have a research position).

____

____

____

____

____

5. Please choose the option below that best describes your research experience.

○ I am working as an undergraduate researcher in a campus lab

○ I am working as an undergraduate researcher in an LBNL lab

○ I am performing research through an established undergraduate research program (i.e. BLUR, URAP). Please include the program below:

____

○ I am or have performed research through an established summer research program (i.e. Amgen scholars, SURF, REU). Please include the program below:

____

○ I am interested in performing research but have not found a position yet.

○ I have performed research in the past but am not currently in a research position

○ I am not interested in performing research

○ Other __________________________________

6. What is your major?

____

7. (If applicable) Approximately how many times did you meet with your TSMP graduate student mentor?

____

8. (If applicable) Did you feel that your TSMP graduate student mentor was well matched to you and your research interests? Please describe why or why not.

____

____

____

____

____

9. (If applicable) Approximately how many TSMP meetings did you attend?

- ○ 1
- ○ 2
- ○ 3
- ○ 4
- ○ 5
- ○ 6

10. (If applicable) Do you have an ongoing relationship with other participants you met through the TSMP?

- ○ Yes
- ○ No
- ○ Other ________________________________

11. (If applicable) Which TSMP seminar(s) did you like best? What about the seminars did you find useful?

____

12. How strongly do you identify with the character on the right in the cartoon?

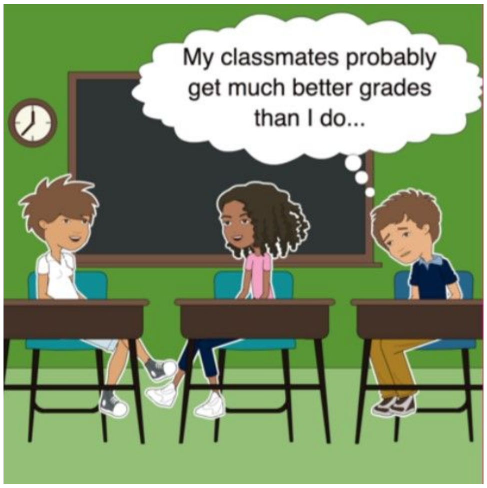

- 0 – Do not identify
- 1
- 2
- 3
- 4
- 5
- 6
- 7
- 8
- 9
- 10 - Very strongly identify

13.

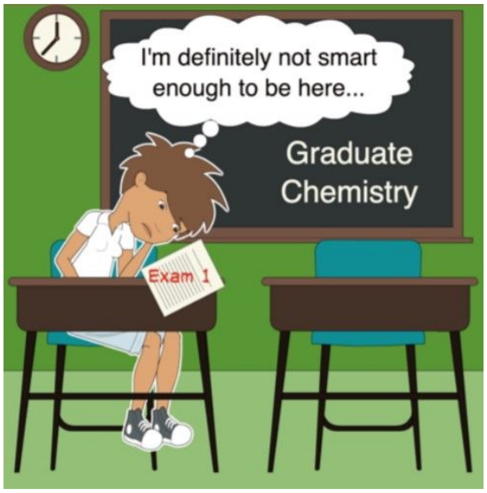

- 0 – Do not identify
- 1
- 2
- 3
- 4
- 5
- 6
- 7
- 8
- 9
- 10 - Very strongly identify

14.

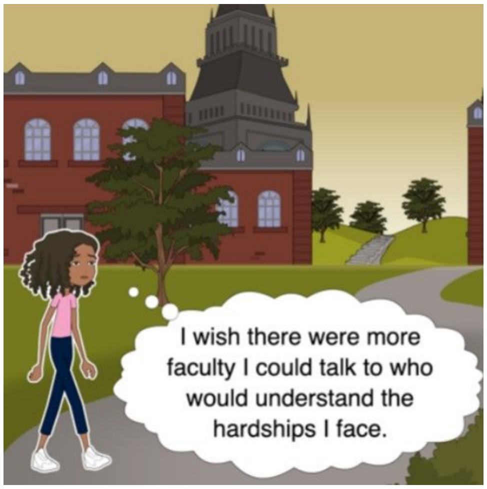

- 0 – Do not identify
- 1
- 2
- 3
- 4
- 5
- 6
- 7
- 8
- 9
- 10 - Very strongly identify

15. How strongly do you identify with the character on the left of the cartoon?

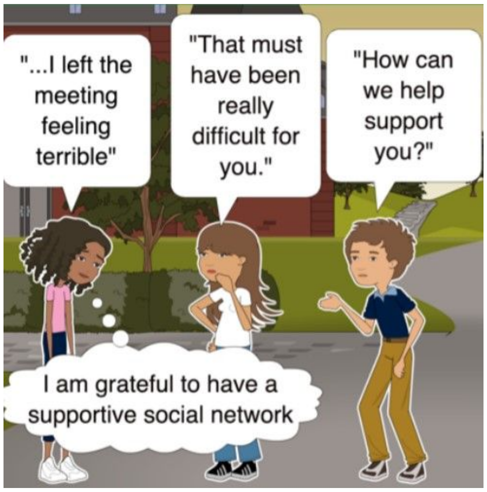

- 0 – Do not identify
- 1
- 2
- 3
- 4
- 5
- 6
- 7
- 8
- 9
- 10 - Very strongly identify

16.

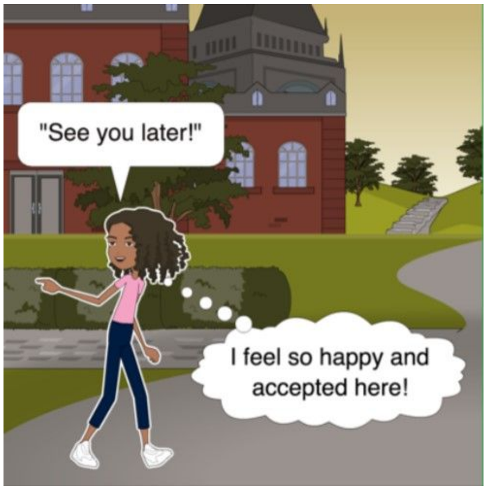

- 0 – Do not identify
- 1
- 2
- 3
- 4
- 5
- 6
- 7
- 8
- 9
- 10 - Very strongly identify

17.

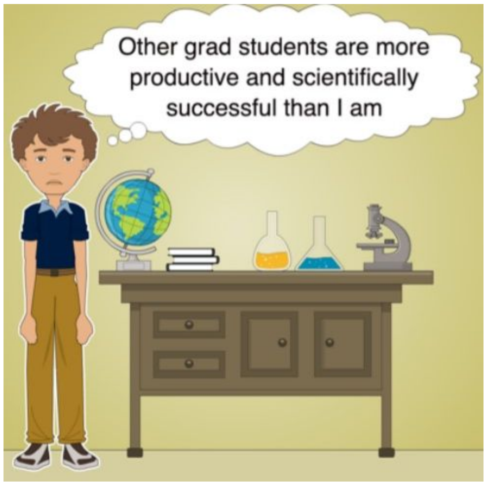

- 0 – Do not identify
- 1
- 2
- 3
- 4
- 5
- 6
- 7
- 8
- 9
- 10 - Very strongly identify

18.

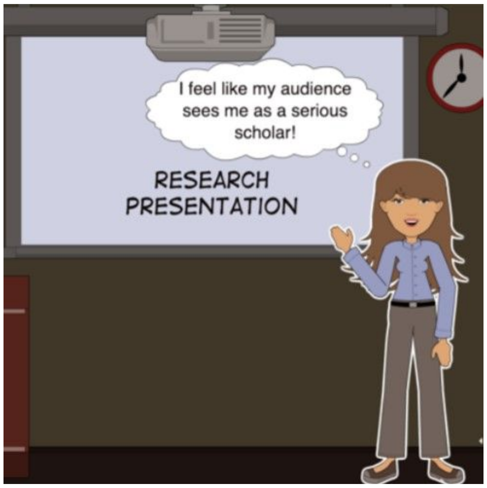

- 0 – Do not identify
- 1
- 2
- 3
- 4
- 5
- 6
- 7
- 8
- 9
- 10 - Very strongly identify

19.

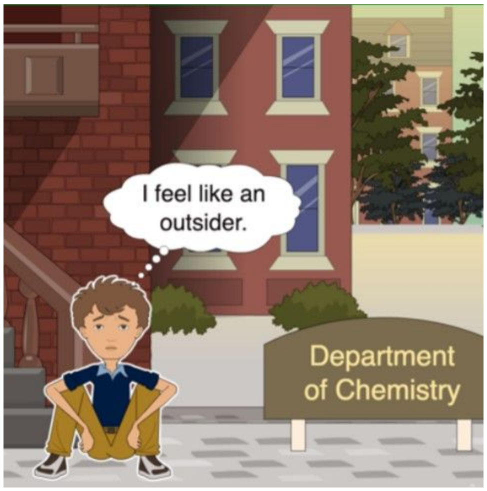

- 0 – Do not identify
- 1
- 2
- 3
- 4
- 5
- 6
- 7
- 8
- 9
- 10 - Very strongly identify

20.

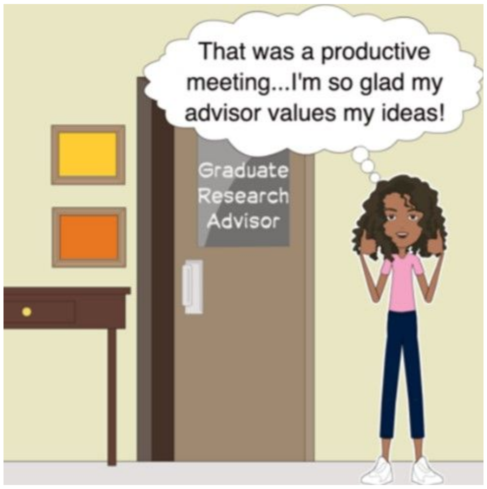

- ◯ 0 – Do not identify
- ◯ 1
- ◯ 2
- ◯ 3
- ◯ 4
- ◯ 5
- ◯ 6
- ◯ 7
- ◯ 8
- ◯ 9
- ◯ 10 - Very strongly identify

21.

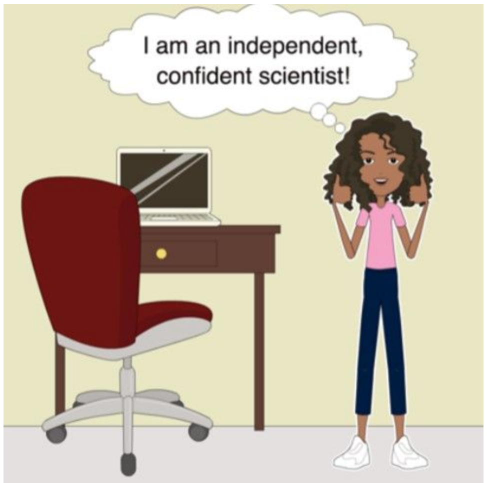

- 0 – Do not identify
- 1
- 2
- 3
- 4
- 5
- 6
- 7
- 8
- 9
- 10 - Very strongly identify

22. Think about what it means to you to feel "at home" somewhere. How does this feeling apply to your experience in the [Departments of Chemistry and Chemical and Biomolecular Engineering at this institution]?

23. What to you constitutes a scientist? Do you describe yourself as a scientist? Why or why not? (For engineering students, please answer these questions for "engineer" rather than "scientist").

Your data will be kept private and only accessible by the study directors via password-protected computers. Please keep in mind that while your data is kept as secure and private as possible, no guarantees can be made against the low risk of an inadvertent breach in confidentiality. For this reason, if there is any risk that a breach of your responses would lead to any damage or discomfort we encourage you to please leave the question blank.

24. (Optional) Please state the gender you best identify with.

25. (Optional) Please state the sexuality you best identify with.

____

26. (Optional) Please state the race/ethnicity you best identify with.

____

27. (Optional) Are you the first in your immediate family to attend college?

- ○ Yes
- ○ No

TSMP Graduate Student Pre-Program Survey (August 2021)

All images used in this survey were reproduced under CC BY International 4.0 License, from ref 1. 2020, *PLOS ONE*.

1. What year are you?

- ○ 1st year
- ○ 2nd year
- ○ 3rd year
- ○ 4th year
- ○ 5th year
- ○ 6th year
- ○ Other ______________________

2. Have you previously worked with the Transfer Student Mentorship Program (TSMP)?

- ○ Yes
- ○ No
- ○ Other ______________________

3. Please briefly describe any previous experience you've had mentoring (can simply list mentorship positions).

____

4. How strongly do you identify with the character on the right in the cartoon?

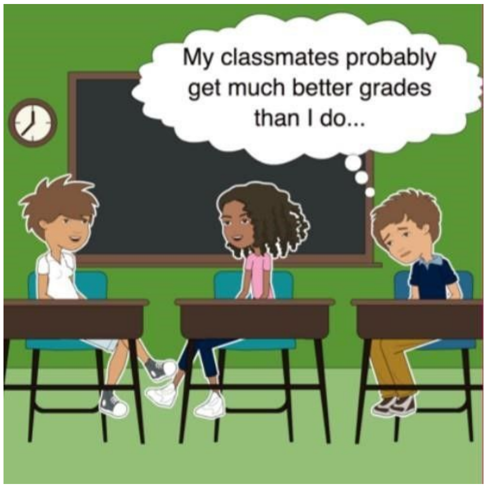

- 0 – Do not identify
- 1
- 2
- 3
- 4
- 5
- 6
- 7
- 8
- 9
- 10 - Very strongly identify

5.

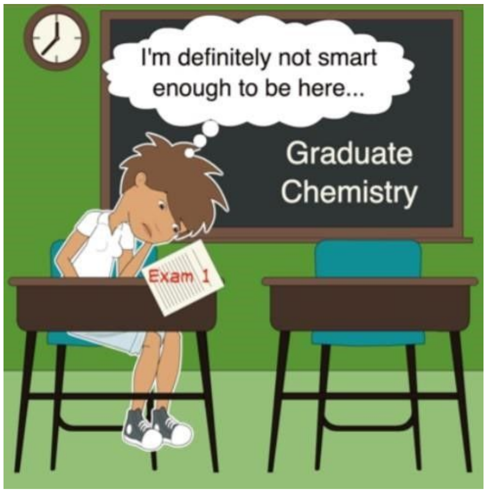

- ◯ 0 – Do not identify
- ◯ 1
- ◯ 2
- ◯ 3
- ◯ 4
- ◯ 5
- ◯ 6
- ◯ 7
- ◯ 8
- ◯ 9
- ◯ 10 - Very strongly identify

6.

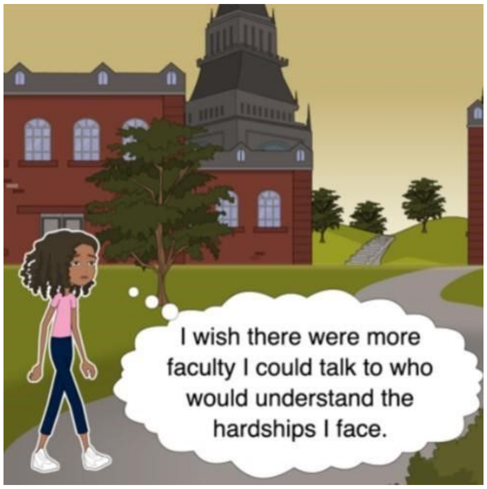

- 0 – Do not identify
- 1
- 2
- 3
- 4
- 5
- 6
- 7
- 8
- 9
- 10 - Very strongly identify

7. How strongly do you identify with the character on the left of the cartoon?

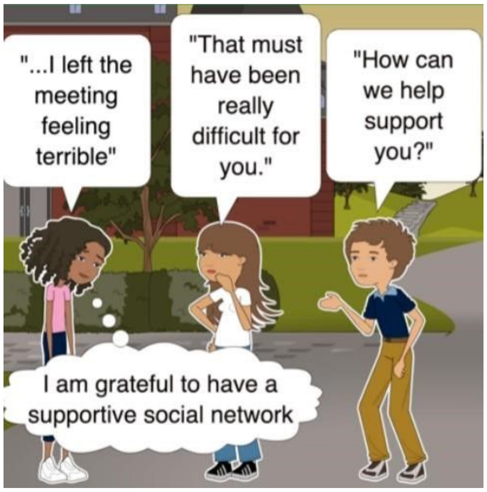

- 0 – Do not identify
- 1
- 2
- 3
- 4
- 5
- 6
- 7
- 8
- 9
- 10 - Very strongly identify

8.

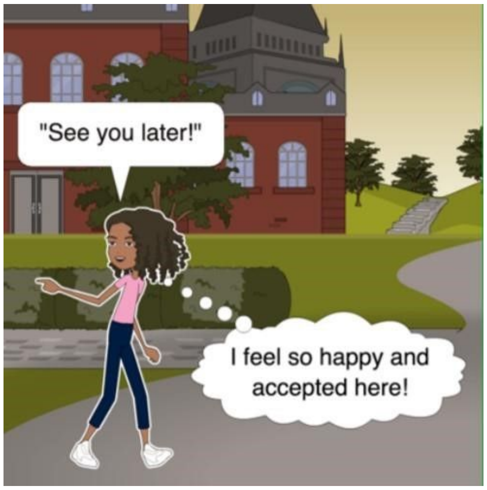

- ◯ 0 – Do not identify
- ◯ 1
- ◯ 2
- ◯ 3
- ◯ 4
- ◯ 5
- ◯ 6
- ◯ 7
- ◯ 8
- ◯ 9
- ◯ 10 - Very strongly identify

9.

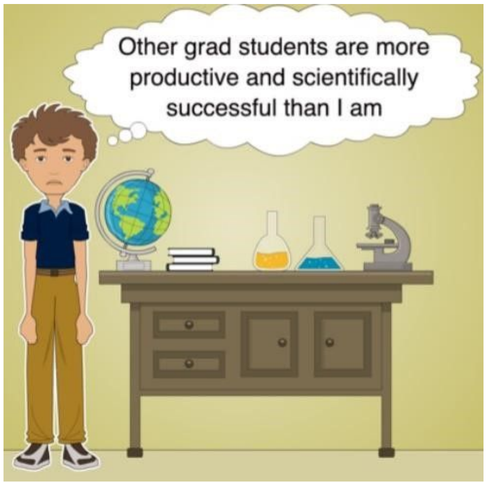

- 0 – Do not identify
- 1
- 2
- 3
- 4
- 5
- 6
- 7
- 8
- 9
- 10 - Very strongly identify

10.

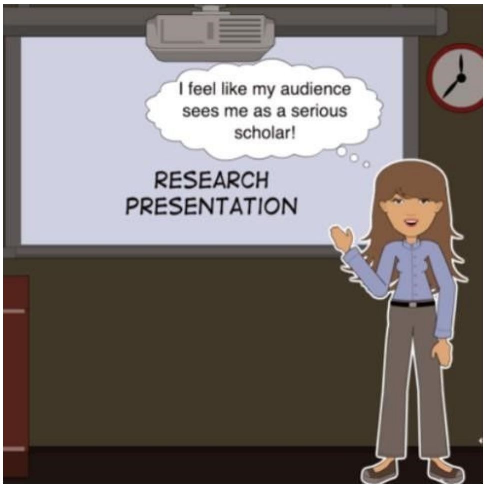

- 0 – Do not identify
- 1
- 2
- 3
- 4
- 5
- 6
- 7
- 8
- 9
- 10 - Very strongly identify

11.

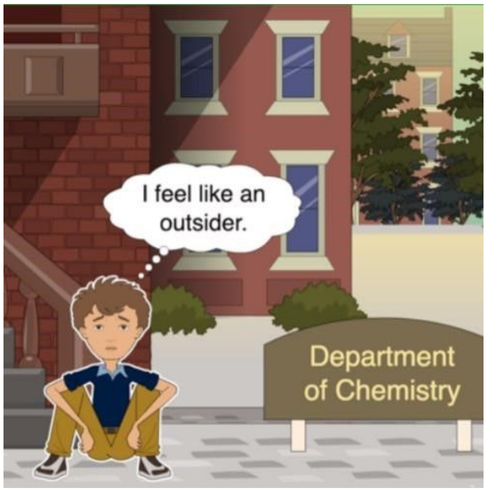

- 0 – Do not identify
- 1
- 2
- 3
- 4
- 5
- 6
- 7
- 8
- 9
- 10 - Very strongly identify

12.

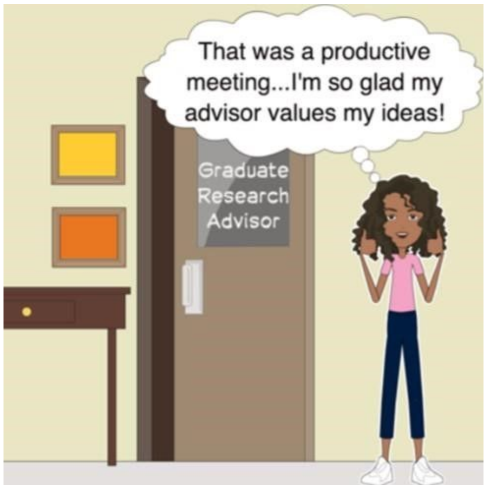

- 0 – Do not identify
- 1
- 2
- 3
- 4
- 5
- 6
- 7
- 8
- 9
- 10 - Very strongly identify

13.

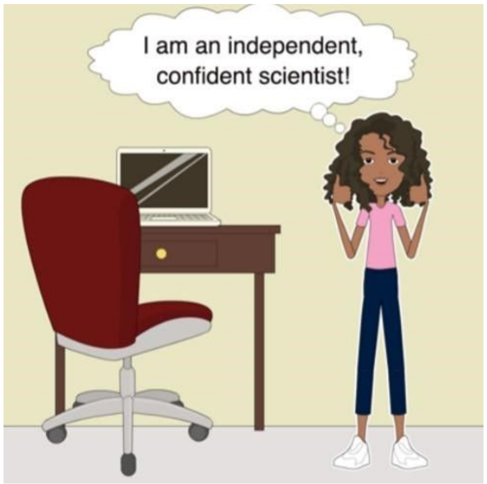

- 0 – Do not identify
- 1
- 2
- 3
- 4
- 5
- 6
- 7
- 8
- 9
- 10 - Very strongly identify

14. Think about what it means to you to feel "at home" somewhere. How does this feeling apply to your experience in the [Departments of Chemistry and Chemical and Biomolecular Engineering at this institution]?

15. What to you constitutes a scientist? Do you describe yourself as a scientist? Why or why not?

Your data will be kept private and only accessible by the study directors via password-protected computers. Please keep in mind that while your data is kept as secure and private as possible, no guarantees can be made against the low risk of an inadvertent breach in confidentiality. For this reason, if there is any risk that a breach of your responses would lead to any damage or discomfort we encourage you to please leave the question blank.

16. (Optional) Please state the gender you best identify with.

17. (Optional) Please state the sexuality you best identify with.

____

18. (Optional) Please state the race/ethnicity you best identify with.

____

19. (Optional) Are you the first in your immediate family to attend college?

- ○ Yes
- ○ No

TSMP Graduate Student Post-Program Survey (December 2021)

All images used in this survey were reproduced under CC BY International 4.0 License, from ref 1. 2020, *PLOS ONE*.

1. What year are you?

- ○ 1st year
- ○ 2nd year
- ○ 3rd year
- ○ 4th year
- ○ 5th year
- ○ 6th year
- ○ Other ______________________________

---

2. Have you previously worked with the Transfer Student Mentorship Program (TSMP)?

- ○ Yes
- ○ No
- ○ Other ______________________________

---

3. Please briefly describe any previous experience you've had mentoring (can simply list mentorship positions).

____

---

4. Approximately how many times did you meet with your transfer student mentee?

____

5. Did you feel that you and your transfer student mentee were well matched? Please outline why or why not.

____

____

____

____

____

6. How many TSMP meetings did you attend?

- ○ 1
- ○ 2
- ○ 3
- ○ 4
- ○ 5
- ○ 6

7. Do you have an ongoing relationship with any other participants you met through the TSMP?

____

8. Which TSMP seminar did you find the most useful and why?

9. What has the impact of the TSMP been on your professional development?

10. How strongly do you identify with the character on the right in the cartoon?

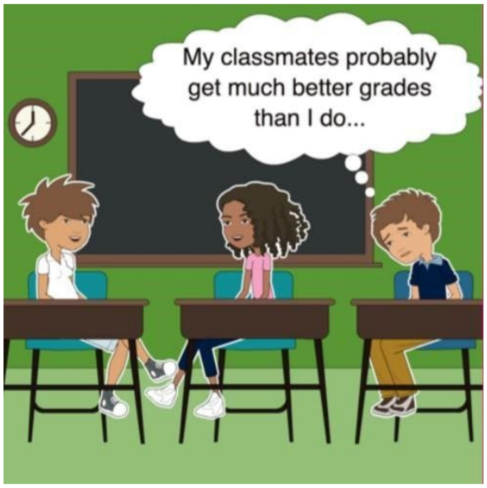

- ◯ 0 – Do not identify
- ◯ 1
- ◯ 2
- ◯ 3
- ◯ 4
- ◯ 5
- ◯ 6
- ◯ 7
- ◯ 8
- ◯ 9
- ◯ 10 - Very strongly identify

11.

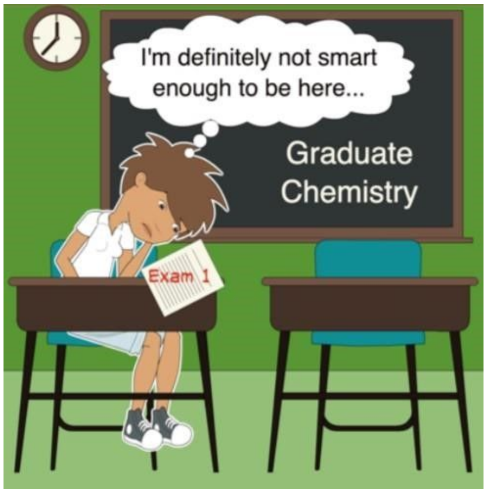

- ○ 0 – Do not identify
- ○ 1
- ○ 2
- ○ 3
- ○ 4
- ○ 5
- ○ 6
- ○ 7
- ○ 8
- ○ 9
- ○ 10 - Very strongly identify

12.

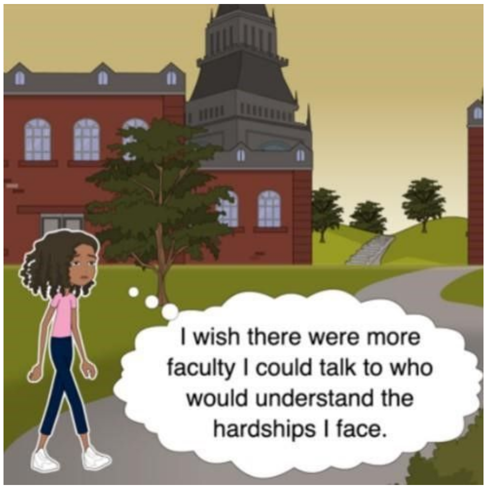

- 0 – Do not identify
- 1
- 2
- 3
- 4
- 5
- 6
- 7
- 8
- 9
- 10 - Very strongly identify

13. How strongly do you identify with the character on the left of the cartoon?

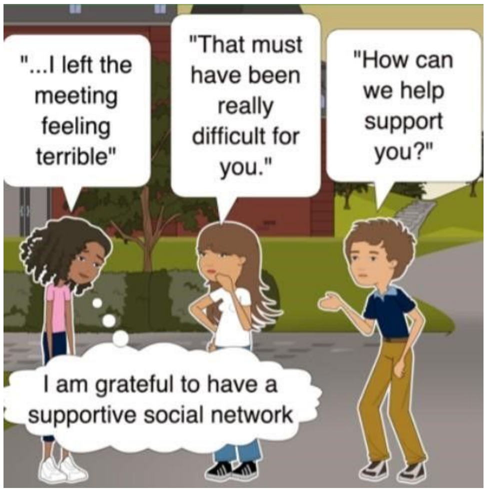

- 0 – Do not identify
- 1
- 2
- 3
- 4
- 5
- 6
- 7
- 8
- 9
- 10 - Very strongly identify

14.

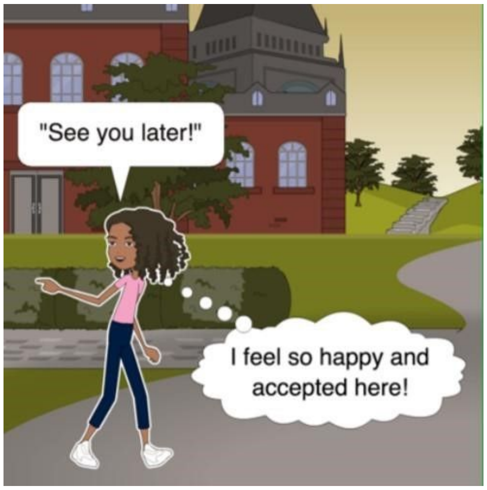

- 0 – Do not identify
- 1
- 2
- 3
- 4
- 5
- 6
- 7
- 8
- 9
- 10 - Very strongly identify

15.

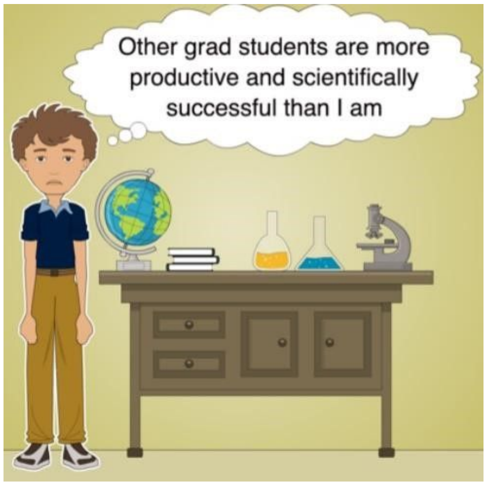

- ◯ 0 – Do not identify
- ◯ 1
- ◯ 2
- ◯ 3
- ◯ 4
- ◯ 5
- ◯ 6
- ◯ 7
- ◯ 8
- ◯ 9
- ◯ 10 - Very strongly identify

16.

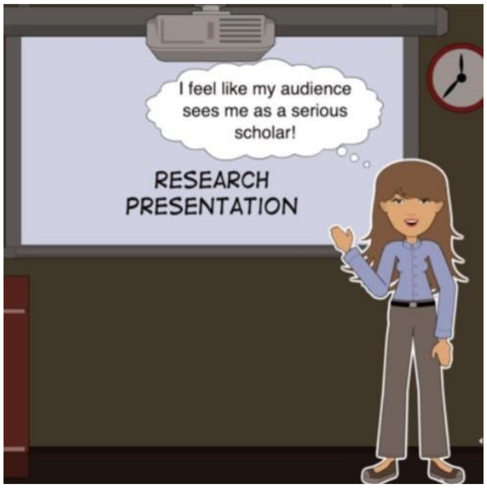

- ◯ 0 – Do not identify
- ◯ 1
- ◯ 2
- ◯ 3
- ◯ 4
- ◯ 5
- ◯ 6
- ◯ 7
- ◯ 8
- ◯ 9
- ◯ 10 - Very strongly identify

17.

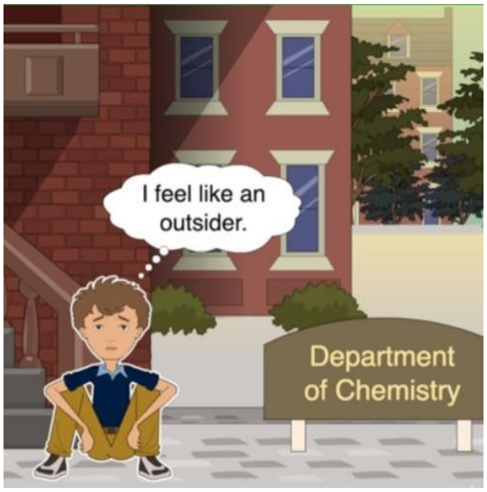

- 0 – Do not identify
- 1
- 2
- 3
- 4
- 5
- 6
- 7
- 8
- 9
- 10 - Very strongly identify

18.

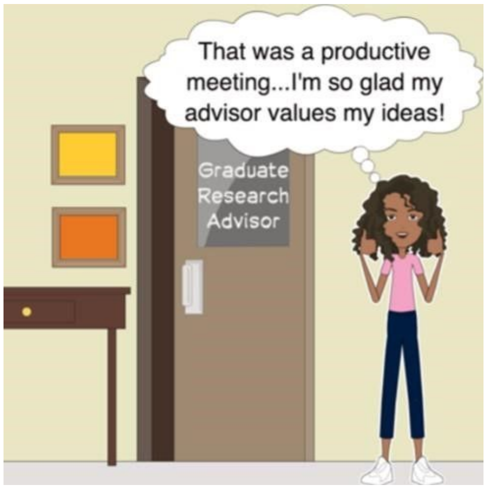

- 0 – Do not identify
- 1
- 2
- 3
- 4
- 5
- 6
- 7
- 8
- 9
- 10 - Very strongly identify

19.

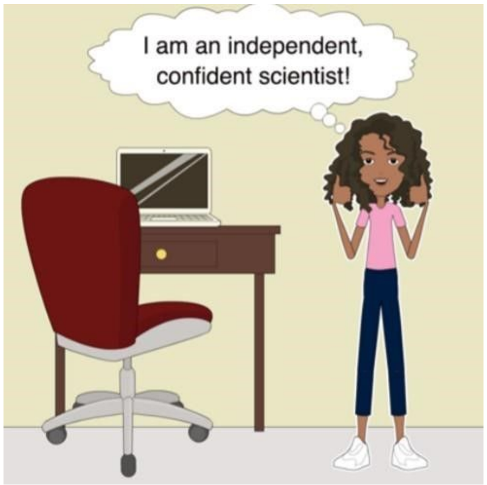

- 0 – Do not identify
- 1
- 2
- 3
- 4
- 5
- 6
- 7
- 8
- 9
- 10 - Very strongly identify

20. Think about what it means to you to feel "at home" somewhere. How does this feeling apply to your experience in the [Departments of Chemistry and Chemical and Biomolecular Engineering at this institution]?

21. What to you constitutes a scientist? Do you describe yourself as a scientist? Why or why not?

Your data will be kept private and only accessible by the study directors via password-protected computers. Please keep in mind that while your data is kept as secure and private as possible, no guarantees can be made against the low risk of an inadvertent breach in confidentiality. For this reason, if there is any risk that a breach of your responses would lead to any damage or discomfort we encourage you to please leave the question blank.

22. (Optional) Please state the gender you best identify with.

23. (Optional) Please state the sexuality you best identify with.

____

24. (Optional) Please state the race/ethnicity you best identify with.

____

25 (Optional) Are you the first in your immediate family to attend college?

- ○ Yes
- ○ No

TSMP Graduate Student Follow-Up Survey (March 2022)

1. To the best of your knowledge, did your first mentee have a research position before the beginning of the program?

- ○ Yes
- ○ No
- ○ I don't know
- ○ Other________________________________

2. To the best of your knowledge, does your first mentee have a research position now?

- ○ Yes
- ○ No
- ○ I don't know
- ○ Other________________________________

3. Approximately how many times did you meet with your first mentee?

- ◯ 0
- ◯ 1
- ◯ 2
- ◯ 3
- ◯ 4
- ◯ 5
- ◯ 6
- ◯ Other____

The following questions are for mentors who had more than one mentee. If you had only one mentee, please skip to the end of the survey and click the forward arrow to finish.

4. To the best of your knowledge, if you had a second mentee, did your second mentee have a research position before the beginning of the program?

- ◯ Yes
- ◯ No
- ◯ I don't know
- ◯ Other____

5. To the best of your knowledge, if you had a second mentee, does your second mentee have a research position now?

- ◯ Yes
- ◯ No
- ◯ I don't know
- ◯ Other________________________________

---

6. If you had a second mentee, approximately how many times did you meet with your second mentee?

- ◯ 0
- ◯ 1
- ◯ 2
- ◯ 3
- ◯ 4
- ◯ 5
- ◯ 6
- ◯ Other________________________________

References

1. Stachl, C. N.; Baranger, A. M. Sense of Belonging within the Graduate Community of a Research-Focused STEM Department: Quantitative Assessment Using a Visual Narrative and Item Response Theory. *PLOS ONE* **2020**, *15 (5)*, 1–27. DOI: 10.1371/journal.pone.0233431.
