# Supplementary material for: A Pilot Graduate Student-Led Near-Peer Mentorship Program for Transfer Students Provides a Supportive Network at an R1 Institution
Source: J Chem Educ. 2022 Nov 10;100(1):134–42. doi: 10.1021/acs.jchemed.2c00427 (PMC9835829; doi:10.1021/acs.jchemed.2c00427)
Supplement: Supplementary file 2 — ed2c00427_si_002.docx [file ed2c00427_si_002.docx]

Supporting Information

**A Pilot Graduate Student-Led Near-Peer Mentorship Program for Transfer Students Provides a Supportive Network at an R1 Institution**

Audrey G. Reeves^*,†,#^, Amanda J. Bischoff^*, †,#,§^, Brice Yates^†^; Daniel D. Brauer^†^; Anne M. Baranger^†,‡^

^†^Department of Chemistry, University of California, Berkeley, CA, USA, 94720

^#^Molecular Biophysics and Integrated Bioimaging Division, Lawrence Berkeley National Laboratories, Berkeley, CA, USA, 94720

^‡^Graduate Group in Science and Mathematics Education, University of California, Berkeley, CA, USA, 94720

*Address correspondence to these authors.

Audrey G. Reeves email: areeves@berkeley.edu

Amanda J. Bischoff email: amanda_bischoff@berkeley.edu

mentorship program, transfer students, minorities in chemistry, public outreach

**The following document includes:**

| Figure S1: Distribution of number of individual mentorship meetings per mentee | 3 |
| --- | --- |
| Figure S2: Research position status of transfer student study participants over time | 4 |
| Figure S3. The institution’s departmental demographics | 5 |
| Figure S4. Summed belongingness scores of graduate student mentors | 6 |
| Table S1: Agreement item numbering for sense of belonging scale | 7 |
| Table S2: August survey levels of agreement with sense of belonging statements on a 1-10 scale | 8 |
| Table S3: December survey levels of agreement with sense of belonging statements on a 1-10 scale | 9 |
| Table S4: Cronbach's α values for sense of belonging scale | 10 |
| Table S5: Transfer student responses to experience question 1 | 11 |
| Table S6: Transfer student responses to experience question 2 | 12 |
| Table S7: Transfer student responses to experience question 3 | 13 |
| Table S8: Transfer student responses to experience question 4 | 14 |
| Table S9: Graduate student responses to experience question 1 | 15 |
| Table S10: Graduate student responses to experience question 2 | 16 |
| Table S11: Graduate student responses to experience question 3 | 17 |
| Table S12: Graduate student responses to experience question 4 | 18 |
| Table S13: Graduate student responses to experience question 5 | 19 |
| TSMP Transfer Student Pre-Program Survey (August 2021) | 20 |
| TSMP Transfer Student Post-Program Survey (December 2021) | 34 |
| TSMP Graduate Student Pre-Program Survey (August 2021) | 50 |
| TSMP Graduate Student Post-Program Survey (December 2021) | 63 |
| TSMP Graduate Student Follow-Up Survey (March 2022) | 78 |
| References | 81 |


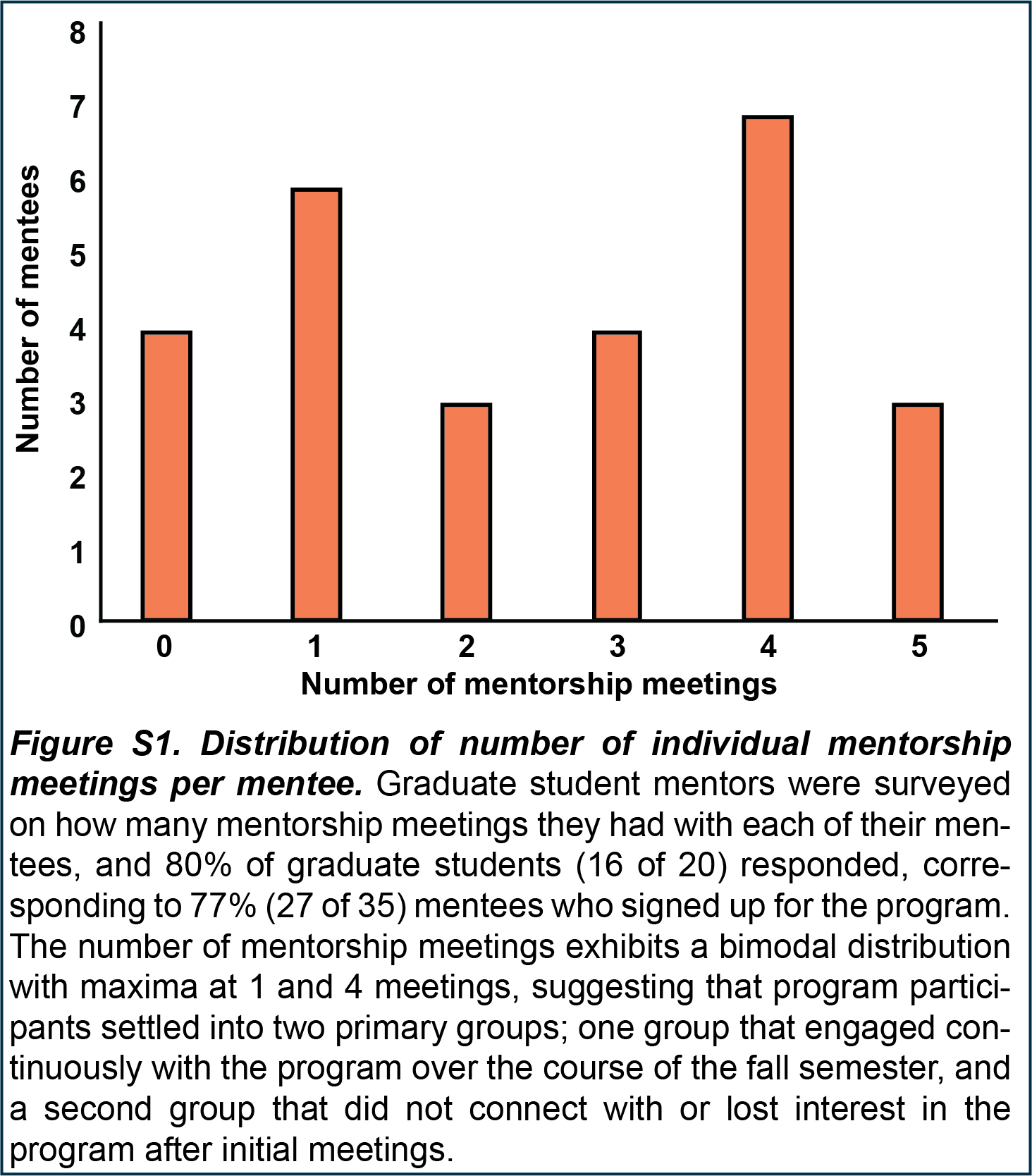


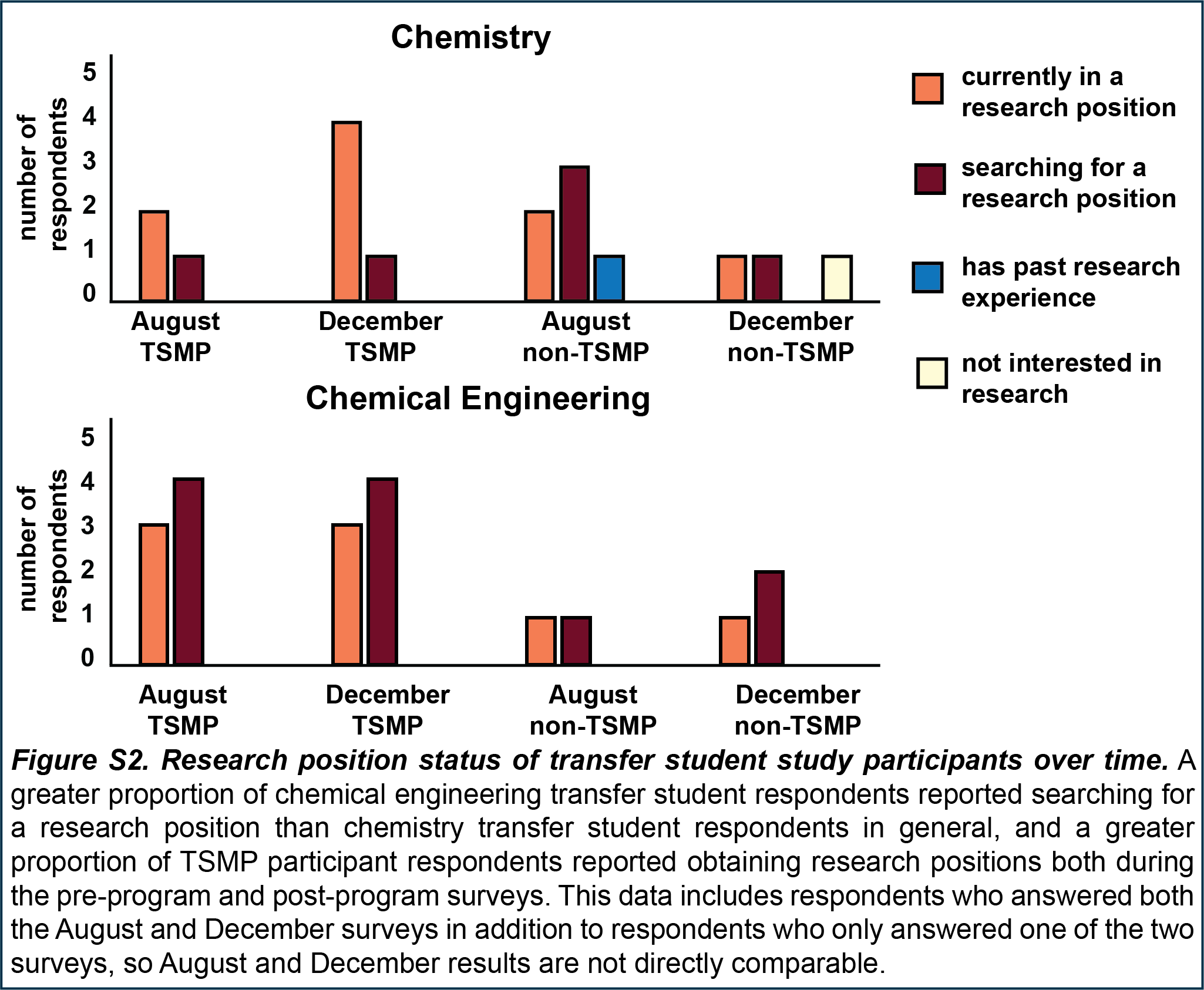


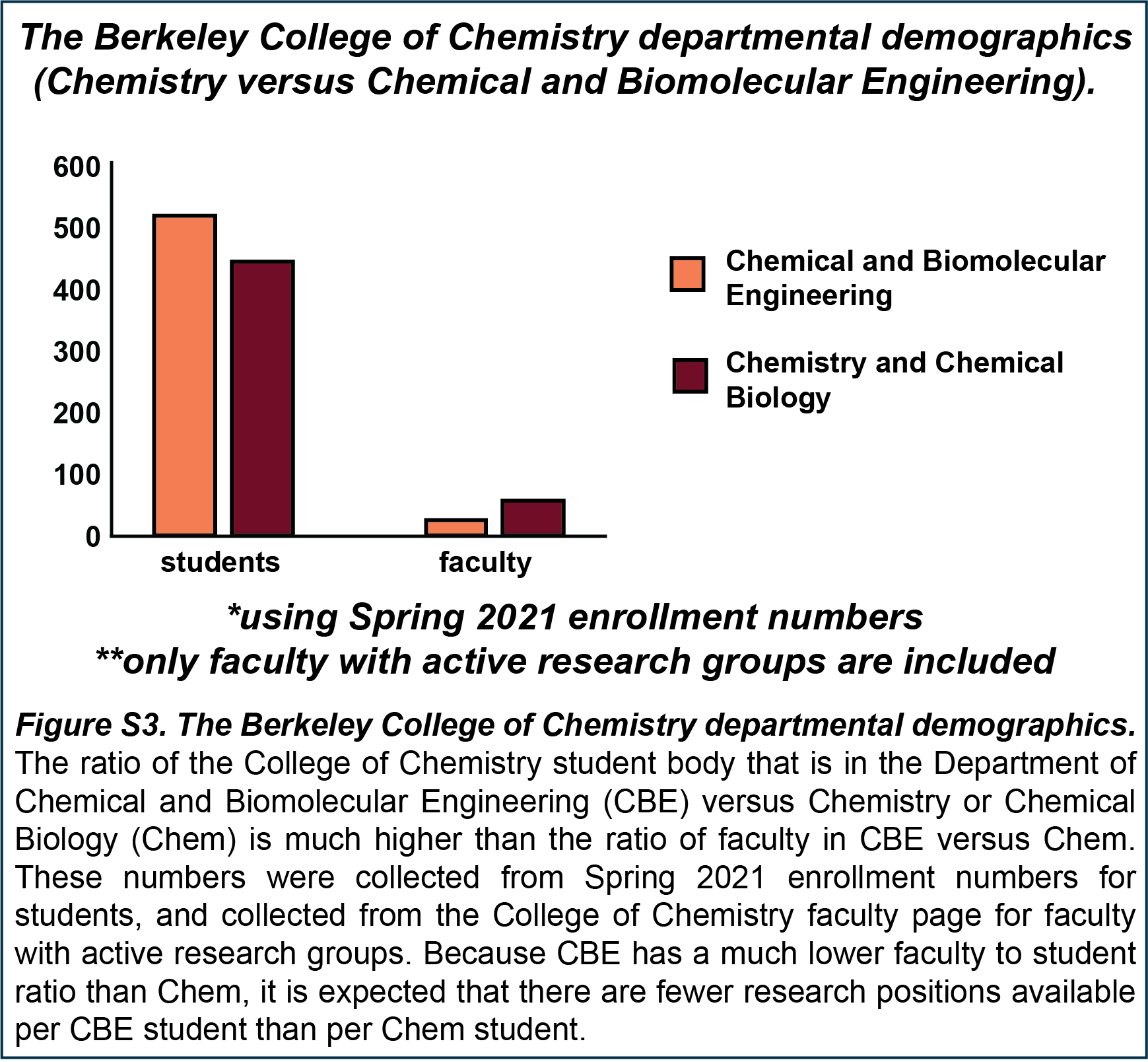


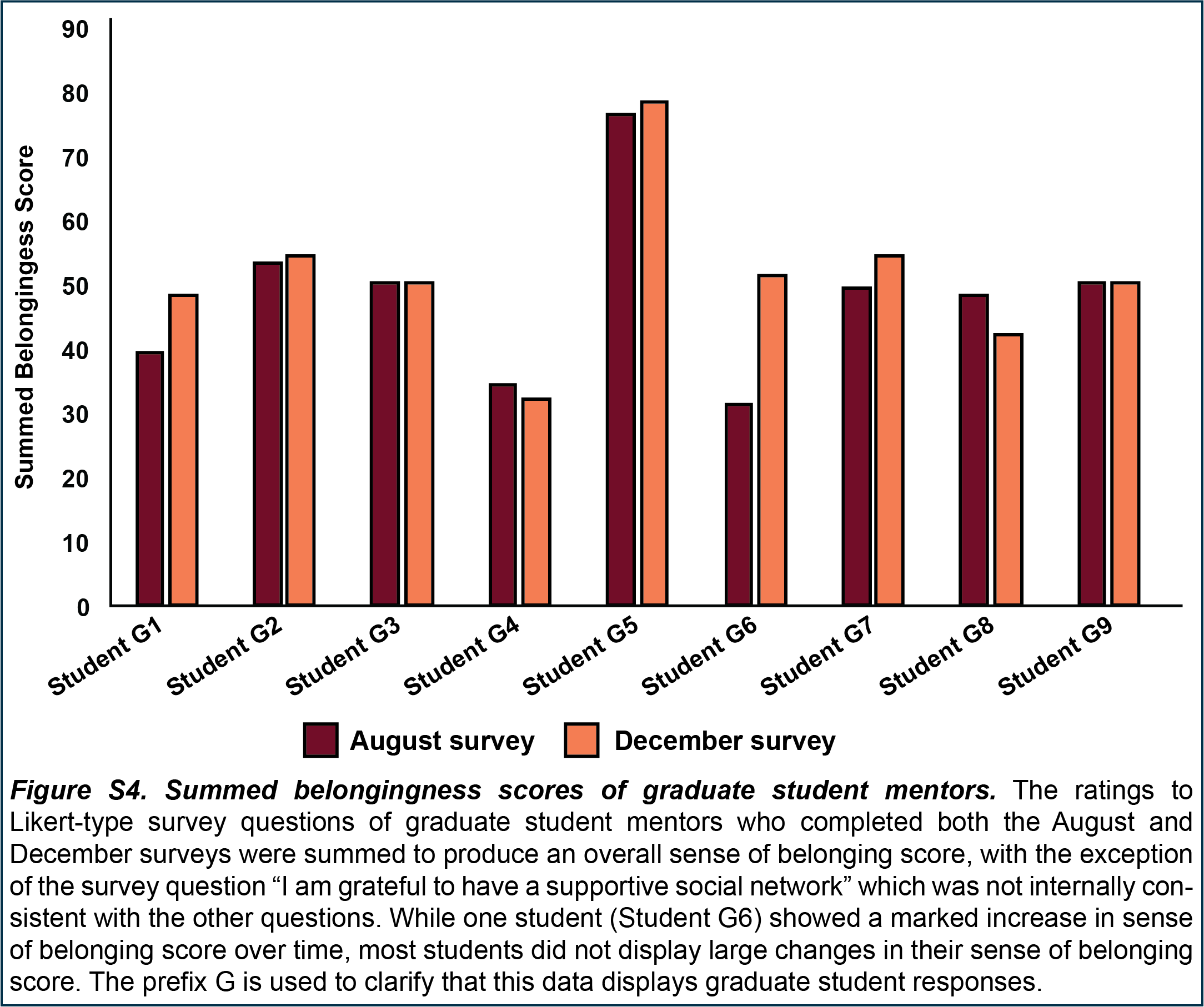


| **Table S1: Agreement item numbering for sense of belonging scale** | | |
| --- | --- | --- |
| Agreement item number | Caption for Transfer Students | Caption for graduate students |
| 1 | My classmates probably get much better grades than I do… | My classmates probably get much better grades than I do… |
| 2 | I'm definitely not smart enough to be here… (in chemistry class) | I'm definitely not smart enough to be here… (in chemistry class) |
| 3 | I wish there were more faculty I could talk to who would understand the hardships I face. | I wish there were more faculty I could talk to who would understand the hardships I face. |
| 4 | I am grateful to have a supportive social network | I am grateful to have a supportive social network |
| 5 | I feel so happy and accepted here! | I feel so happy and accepted here! |
| 6 | Other students are more productive and scientifically successfully than I am | Other grad students are more productive and scientifically successfully than I am |
| 7 | I feel like my audience sees me as a serious scholar! | I feel like my audience sees me as a serious scholar! |
| 8 | I feel like an outsider. (next to chemistry building) | I feel like an outsider. (next to chemistry building) |
| 9 | My instructor values my ideas and respects me. | That was a productive meeting… I'm so glad my advisor values my ideas! |
| 10 | I am an independent, confident scientist! | I am an independent, confident scientist! |

| **Table S2: August survey levels of agreement with sense of belonging statements on a 1-10 scale** | | | | | | | | | | |
| --- | --- | --- | --- | --- | --- | --- | --- | --- | --- | --- |
| Agreement Item | 1 | 2 | 3 | 4 | 5 | 6 | 7 | 8 | 9 | 10 |
| Student T1* | 6 | 6 | 6 | 3 | 3 | 6 | 3 | 6 | 3 | 3 |
| Student T2 | 6 | 6 | 5 | 4 | 6 | 4 | 6 | 3 | 8 | 7 |
| Student T3 | 1 | 1 | 1 | 8 | 10 | 3 | 8 | 0 | 8 | 10 |
| Student T4 | 8 | 7 | 8 | 7 | 7 | 6 | 5 | 5 | 7 | 6 |
| Student T5 | 7 | 7 | 8 | 7 | 6 | 8 | 6 | 8 | 8 | 3 |
| Student T6 | 10 | 10 | 10 | 10 | 5 | 10 | 0 | 5 | 10 | 5 |
| Student T7 | 8 | 7 | 8 | 7 | 4 | 7 | 5 | 8 | 5 | 3 |
| Student T8 | 8 | 7 | 3 | 9 | 9 | 7 | 9 | 0 | 8 | 9 |
| Student T9 | 8 | 8 | 5 | 9 | 8 | 9 | 6 | 7 | 8 | 7 |
| Student T10 | 10 | 10 | 10 | 10 | 10 | 10 | 10 | 10 | 10 | 10 |
| Student T11 | 5 | 0 | 1 | 10 | 5 | 5 | 5 | 0 | 10 | 10 |
| Student T12 | 0 | 0 | 0 | 8 | 9 | 0 | 9 | 2 | 8 | 9 |
| Student T13 | 10 | 10 | 8 | 7 | 6 | 8 | 4 | 6 | 5 | 4 |
| Student T14 | 10 | 10 | 7 | 3 | 3 | 8 | 3 | 9 | 5 | 4 |
| Student T15 | 3 | 5 | 4 | 7 | 6 | 4 | - | 3 | 7 | 6 |
| Student T16 | 6 | 6 | 3 | 10 | 4 | 6 | 7 | 4 | 6 | 8 |
| Student T17 | 9 | 10 | 4 | 7 | 10 | 10 | 7 | 6 | 7 | 3 |
| Student T18 | 8 | 8 | 5 | 6 | 7 | 9 | 4 | 5 | 5 | 3 |
| Student G1** | 7 | 7 | 4 | 5 | 5 | 7 | 4 | 5 | 5 | 5 |
| Student G2 | 1 | 4 | 4 | 8 | 8 | 7 | 5 | 4 | 7 | 3 |
| Student G3 | 7 | 5 | 5 | 8 | 8 | 8 | 6 | 3 | 7 | 7 |
| Student G4 | 7 | 7 | 6 | 6 | 5 | 10 | 3 | 7 | 7 | 6 |
| Student G5 | 0 | 0 | 3 | 10 | 10 | 1 | 4 | 0 | 9 | 7 |
| Student G6 | 5 | 7 | 7 | 4 | 2 | 7 | 4 | 7 | 4 | 4 |
| Student G7 | 7 | 7 | 7 | 6 | 7 | - | 8 | 5 | 10 | 7 |
| Student G8 | 3 | 6 | 2 | 8 | 7 | 10 | 7 | 5 | 4 | 7 |
| Student G9 | 7 | 7 | 3 | 8 | 6 | 7 | 7 | 3 | 7 | 5 |
| Student G10 | 3 | 2 | 6 | 6 | 7 | 8 | 5 | 5 | 6 | 6 |
| Student G11 | 6 | 7 | 5 | 8 | 7 | 9 | 4 | 5 | 7 | 6 |
| Student G12 | 5 | 5 | 2 | 7 | 3 | 7 | 4 | 2 | 3 | 6 |
| Student G13 | 2 | 1 | 3 | 7 | 7 | 6 | 5 | 1 | 5 | 5 |

* “T” prefix stands for transfer student

** “G” prefix stands for graduate student

| **Table S3. December survey levels of agreement with sense of belonging statements on a 1-10 scale** | | | | | | | | | | |
| --- | --- | --- | --- | --- | --- | --- | --- | --- | --- | --- |
| Agreement Item | 1 | 2 | 3 | 4 | 5 | 6 | 7 | 8 | 9 | 10 |
| Student T1* | 9 | 9 | 9 | 5 | 1 | 8 | 2 | 10 | 2 | 1 |
| Student T2 | 6 | 7 | 7 | 5 | 6 | 7 | 6 | 4 | 5 | 5 |
| Student T3 | 2 | 0 | 0 | 10 | 10 | 2 | 10 | 0 | 10 | 10 |
| Student T4 | 8 | 8 | 8 | 6 | 6 | 6 | 5 | 7 | 7 | 7 |
| Student T5 | 10 | 10 | 10 | 8 | 4 | 9 | 0 | 9 | 4 | 0 |
| Student T6 | 10 | 10 | 10 | 5 | 5 | 10 | 2 | 7 | 10 | 5 |
| Student T7 | 7 | 0 | 8 | 3 | 2 | 8 | 6 | 7 | 7 | 4 |
| Student T8 | 8 | 6 | 8 | 9 | 9 | 2 | 10 | 0 | 8 | 10 |
| Student T9 | 7 | 5 | 3 | 8 | 7 | 6 | 8 | 5 | 7 | 7 |
| Student T20 | 4 | 2 | 0 | 7 | 8 | 3 | 5 | 1 | 7 | 6 |
| Student T21 | 4 | 3 | 3 | 5 | 7 | 4 | 5 | 3 | 10 | 6 |
| Student T22 | 3 | 3 | 3 | 3 | 3 | 3 | 3 | 3 | 5 | 5 |
| Student T23 | 10 | 7 | 8 | 7 | 8 | 10 | 3 | 9 | 9 | 7 |
| Student T24 | 4 | 4 | 4 | 8 | 8 | 4 | - | - | 8 | 8 |
| Student T25 | 3 | 0 | 10 | 0 | 0 | 0 | 10 | 5 | 2 | 10 |
| Student T26 | 9 | 10 | 10 | 3 | 2 | 9 | 2 | 9 | 9 | 6 |
| Student G1** | 5 | 5 | 2 | 8 | 5 | 10 | 6 | 3 | 7 | 5 |
| Student G2 | 0 | 5 | 7 | 8 | 8 | 7 | 7 | 2 | 7 | 3 |
| Student G3 | 7 | 7 | 3 | 7 | 7 | 8 | 6 | 3 | 8 | 7 |
| Student G4 | 8 | 9 | 6 | 9 | 7 | 9 | 2 | 7 | 8 | 4 |
| Student G5 | 1 | 0 | 3 | 5 | 9 | 3 | 7 | 1 | 10 | 10 |
| Student G6 | 0 | 6 | 6 | 3 | 4 | 2 | 6 | 7 | 6 | 6 |
| Student G7 | 8 | 8 | 8 | 7 | 7 | 4 | 8 | 8 | 10 | 6 |
| Student G8 | 2 | 6 | 2 | 8 | 8 | 10 | 7 | 3 | 6 | 6 |
| Student G9 | 6 | 6 | 6 | 8 | 5 | 6 | 6 | 5 | 6 | 4 |
| Student G10 | 3 | 2 | 7 | 7 | 7 | 9 | 4 | 3 | 8 | 5 |
| Student G14 | 6 | 8 | 4 | 9 | 9 | 10 | 8 | 6 | 7 | 6 |
| Student G15 | 3 | 5 | 2 | 8 | 8 | 6 | 6 | 3 | 8 | 7 |

* “T” prefix stands for transfer student

** “G” prefix stands for graduate student

| **Table S4. Cronbach's α values for sense of belonging scale** | | |
| --- | --- | --- |
| Agreement items | Population | Cronbach's α |
| 1-10 | Graduate students August survey | 0.86 |
| 1-10 | Graduate students December survey | 0.62 |
| 1-10 | Transfer students August survey | 0.89 |
| 1-10 | Transfer students December survey | 0.88 |
| 1-3, 5-10 | Graduate students August survey | 0.83 |
| 1-3, 5-10 | Graduate students December survey | 0.72 |
| 1-3, 5-10 | Transfer students August survey | 0.90 |
| 1-3, 5-10 | Transfer students December survey | 0.90 |

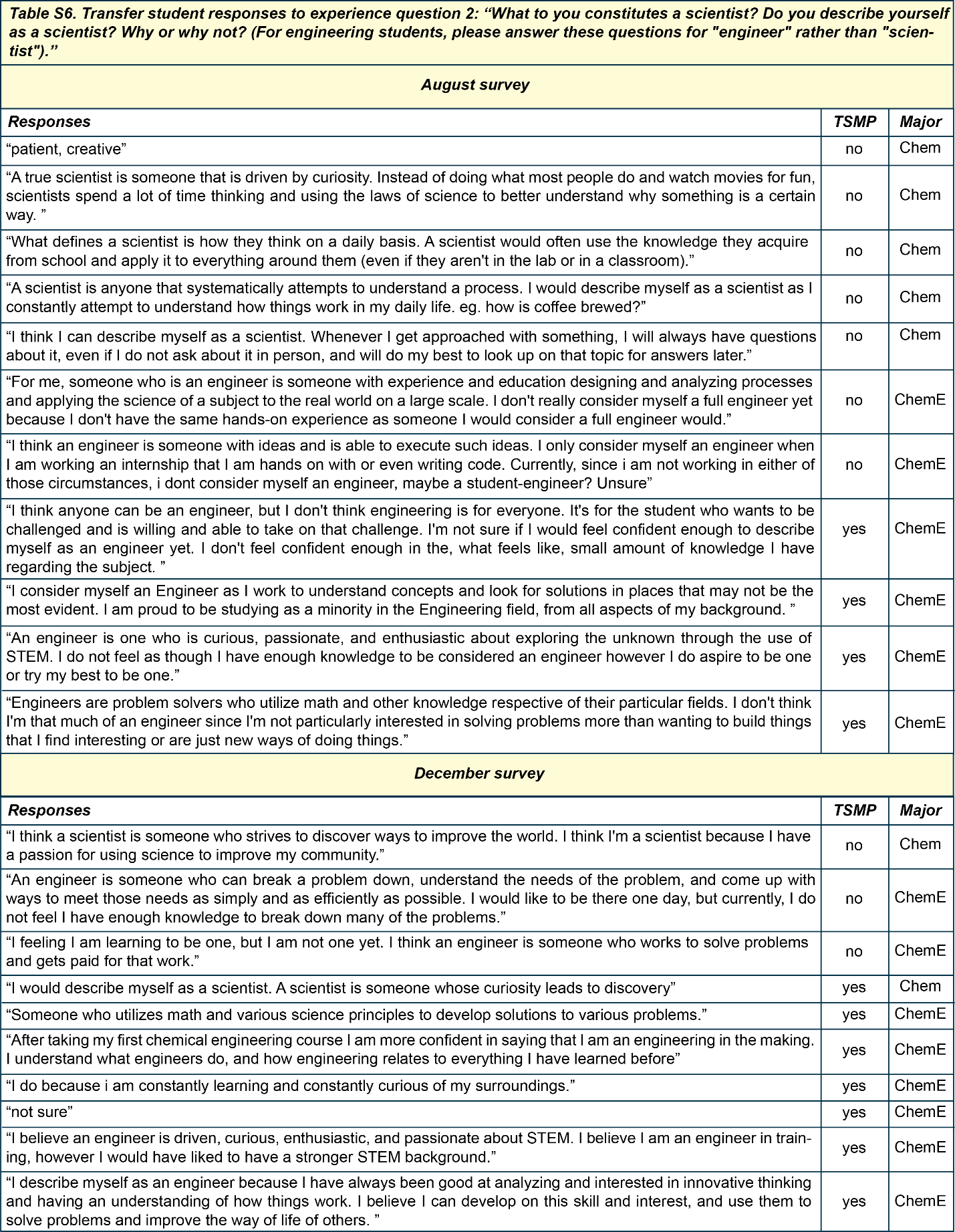


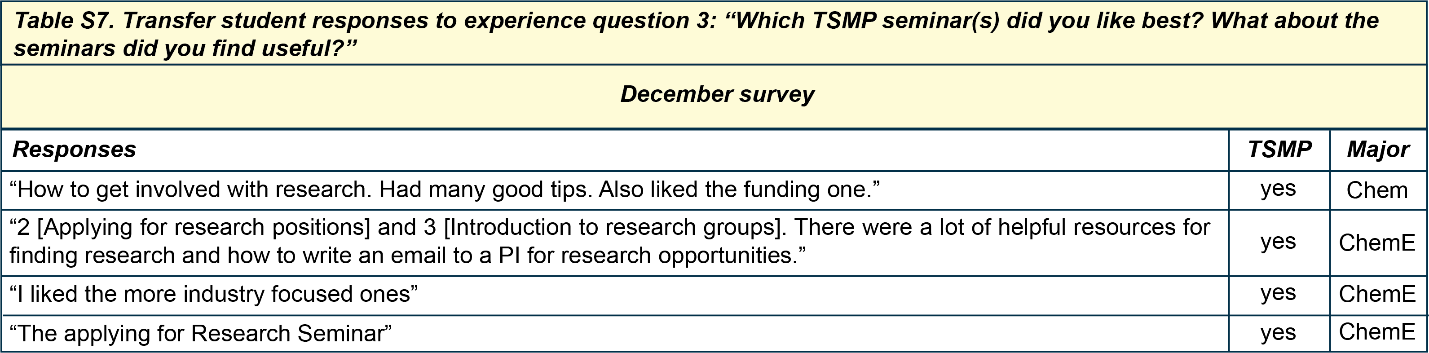

TSMP Transfer Student Pre-Program Survey (August 2021)

All images used in this survey were reproduced under CC BY International 4.0 License, from ref 1. **2020**, *PLOS ONE*.

1. What year are you?

- Sophomore
- Junior
- Senior
- Other _______________________________________________

2. What is your major?

________________________________________________________________

3. Have you ever been involved as an active member of the Transfer Student Mentorship Program (TSMP)?

- Yes
- No
- Other ________________________________________________

4. If you were involved in the TSMP, which year did you first join?

- 2020
- 2021

5. Please describe how you would go about applying for an undergraduate research position in a campus lab (please answer even if you already have a research position).

________________________________________________________________

________________________________________________________________

________________________________________________________________

________________________________________________________________

________________________________________________________________

6. Please choose the option below that best describes your research experience.

- I am working as an undergraduate researcher in a campus lab
- I am working as an undergraduate researcher in an LBNL lab
- I am performing research through an established undergraduate research program (i.e. BLUR, URAP). Please include the program below: ________________________________________________
- I am or have performed research through an established summer research program (i.e. Amgen scholars, SURF, REU). Please include the program below: ________________________________________________
- I am interested in performing research but have not found a position yet.
- I have performed research in the past but am not currently in a research position
- I am not interested in performing research
- Other ________________________________________________

7. How strongly do you identify with the character on the right in the cartoon?


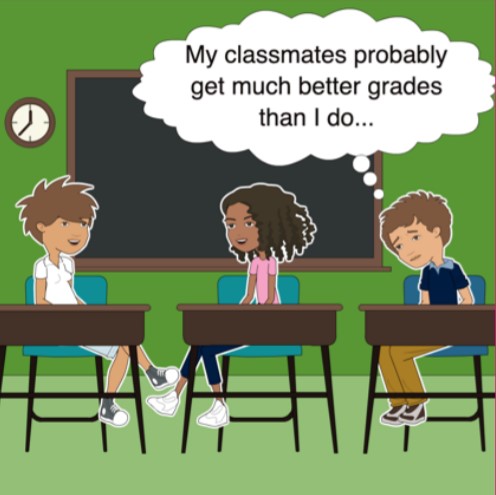


- 0 – Do not identify
- 1
- 2
- 3
- 4
- 5
- 6
- 7
- 8
- 9
- 10 - Very strongly identify

8.


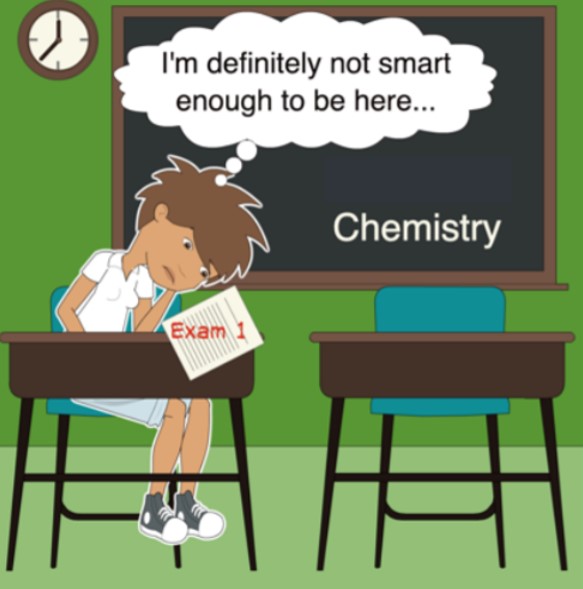


- 0 – Do not identify
- 1
- 2
- 3
- 4
- 5
- 6
- 7
- 8
- 9
- 10 - Very strongly identify

9.


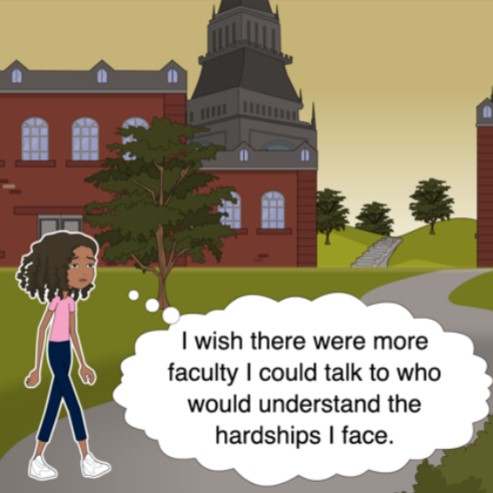


- 0 – Do not identify
- 1
- 2
- 3
- 4
- 5
- 6
- 7
- 8
- 9
- 10 - Very strongly identify

10. How strongly do you identify with the character on the left of the cartoon?


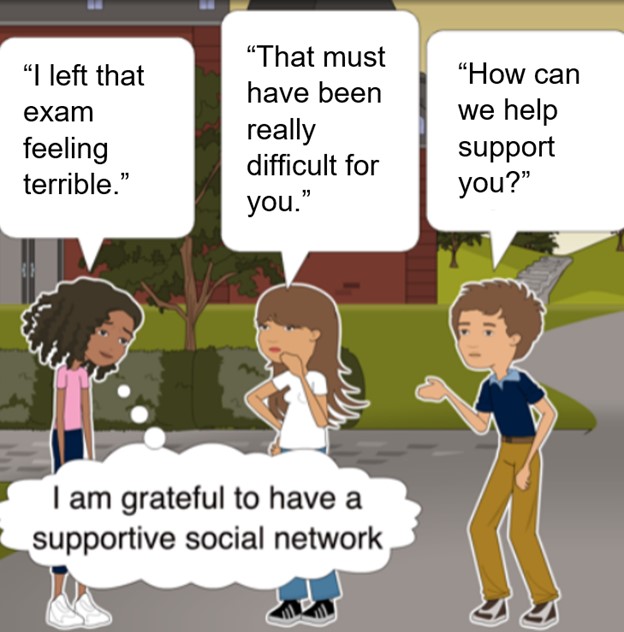


- 0 – Do not identify
- 1
- 2
- 3
- 4
- 5
- 6
- 7
- 8
- 9
- 10 - Very strongly identify

11.


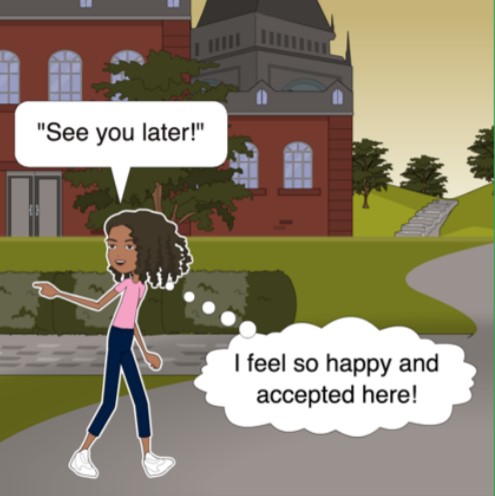


- 0 – Do not identify
- 1
- 2
- 3
- 4
- 5
- 6
- 7
- 8
- 9
- 10 - Very strongly identify

12.


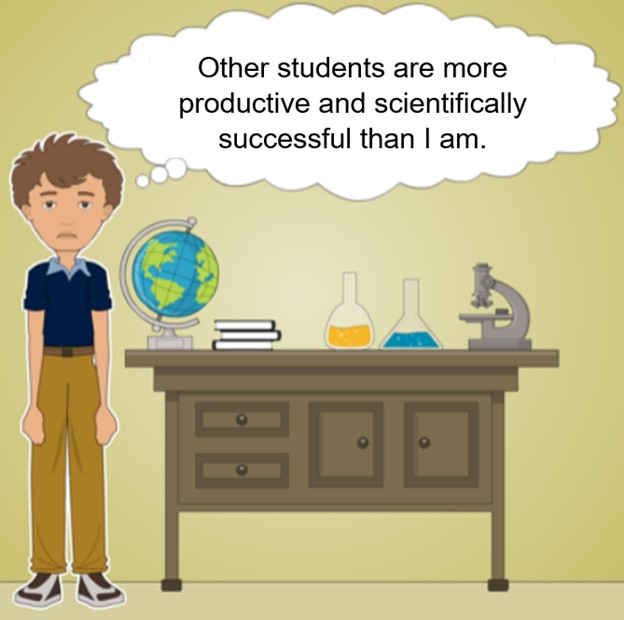


- 0 – Do not identify
- 1
- 2
- 3
- 4
- 5
- 6
- 7
- 8
- 9
- 10 - Very strongly identify

13.


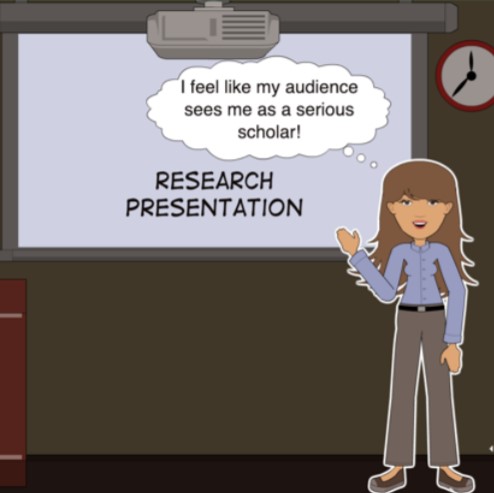


- 0 – Do not identify
- 1
- 2
- 3
- 4
- 5
- 6
- 7
- 8
- 9
- 10 - Very strongly identify

14.


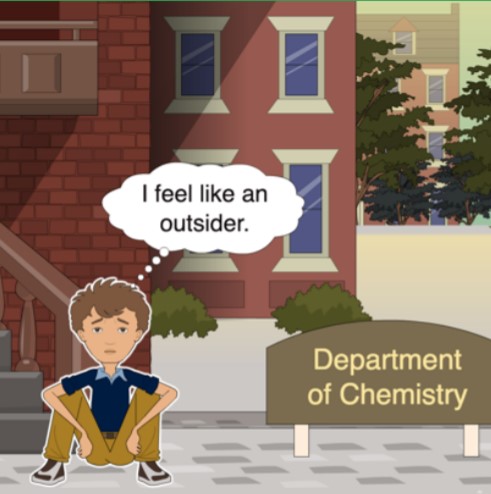


- 0 – Do not identify
- 1
- 2
- 3
- 4
- 5
- 6
- 7
- 8
- 9
- 10 - Very strongly identify

15.


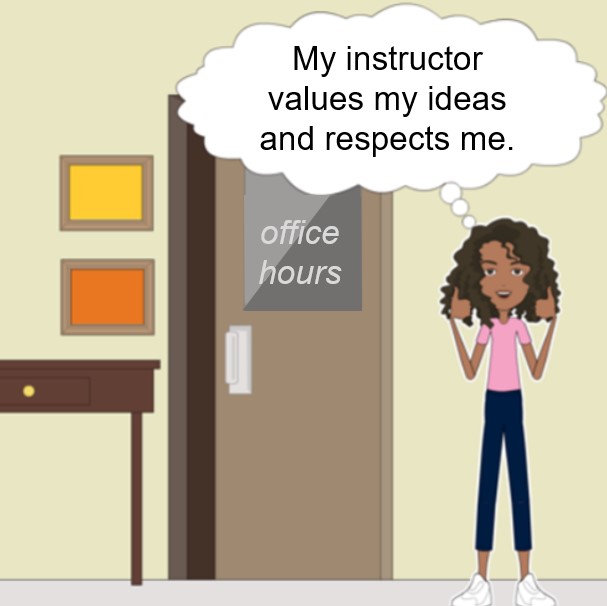


- 0 – Do not identify
- 1
- 2
- 3
- 4
- 5
- 6
- 7
- 8
- 9
- 10 - Very strongly identify

16.


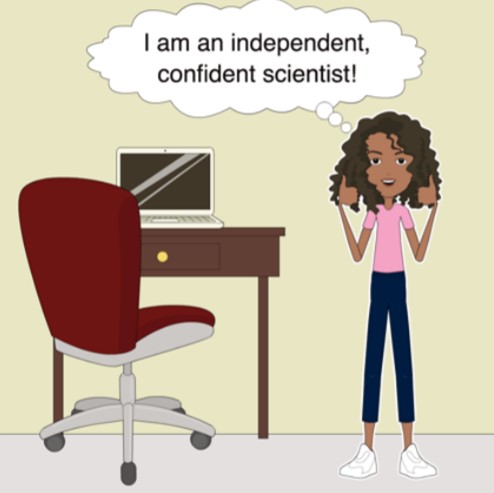


- 0 – Do not identify
- 1
- 2
- 3
- 4
- 5
- 6
- 7
- 8
- 9
- 10 - Very strongly identify

17. Think about what it means to you to feel "at home" somewhere. How does this feeling apply to your experience in the [Departments of Chemistry and Chemical and Biomolecular Engineering at this institution]?

________________________________________________________________

________________________________________________________________

________________________________________________________________

________________________________________________________________

________________________________________________________________

18. What to you constitutes a scientist? Do you describe yourself as a scientist? Why or why not? (For engineering students, please answer these questions for "engineer" rather than "scientist").

________________________________________________________________

________________________________________________________________

________________________________________________________________

________________________________________________________________

________________________________________________________________

Your data will be kept private and only accessible by the study directors via password-protected computers. Please keep in mind that while your data is kept as secure and private as possible, no guarantees can be made against the low risk of an inadvertent breach in confidentiality. For this reason, if there is any risk that a breach of your responses would lead to any damage or discomfort we encourage you to please leave the question blank.

19. (Optional) Please state the gender you best identify with.

________________________________________________________________

20. (Optional) Please state the sexuality you best identify with.

________________________________________________________________

21. (Optional) Please state the race/ethnicity you best identify with.

________________________________________________________________

22. (Optional) Are you the first in your immediate family to attend college?

- Yes
- No

TSMP Transfer Student Post-Program Survey (December 2021)

All images used in this survey were reproduced under CC BY International 4.0 License, from ref 1. **2020**, *PLOS ONE*.

1. What year are you?

- Sophomore
- Junior
- Senior
- Other ________________________________________________

2. Have you ever been involved as an active member of the Transfer Student Mentorship Program (TSMP)?

- Yes
- No
- Other ________________________________________________

3. If you were involved in the TSMP, which year did you first join?

- 2020
- 2021

4. Please describe how you would go about applying for an undergraduate research position in a campus lab (please answer even if you already have a research position).

________________________________________________________________

________________________________________________________________

________________________________________________________________

________________________________________________________________

________________________________________________________________

5. Please choose the option below that best describes your research experience.

- I am working as an undergraduate researcher in a campus lab
- I am working as an undergraduate researcher in an LBNL lab
- I am performing research through an established undergraduate research program (i.e. BLUR, URAP). Please include the program below: ________________________________________________
- I am or have performed research through an established summer research program (i.e. Amgen scholars, SURF, REU). Please include the program below: ________________________________________________
- I am interested in performing research but have not found a position yet.
- I have performed research in the past but am not currently in a research position
- I am not interested in performing research
- Other ________________________________________________

6. What is your major?

________________________________________________________________

7. (If applicable) Approximately how many times did you meet with your TSMP graduate student mentor?

________________________________________________________________

8. (If applicable) Did you feel that your TSMP graduate student mentor was well matched to you and your research interests? Please describe why or why not.

________________________________________________________________

________________________________________________________________

________________________________________________________________

________________________________________________________________

________________________________________________________________

9. (If applicable) Approximately how many TSMP meetings did you attend?

- 1
- 2
- 3
- 4
- 5
- 6

10. (If applicable) Do you have an ongoing relationship with other participants you met through the TSMP?

- Yes
- No
- Other ________________________________________________

11. (If applicable) Which TSMP seminar(s) did you like best? What about the seminars did you find useful?

________________________________________________________________

12. How strongly do you identify with the character on the right in the cartoon?


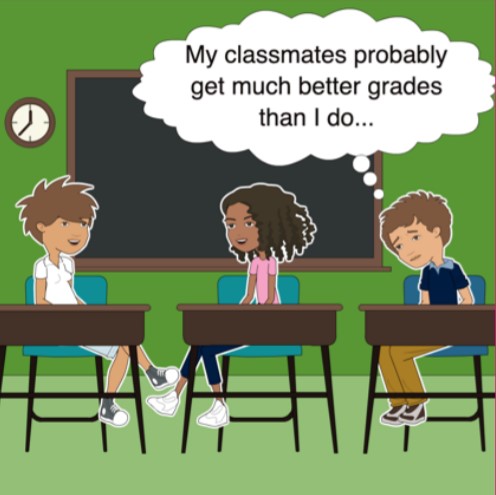


- 0 – Do not identify
- 1
- 2
- 3
- 4
- 5
- 6
- 7
- 8
- 9
- 10 - Very strongly identify

13.


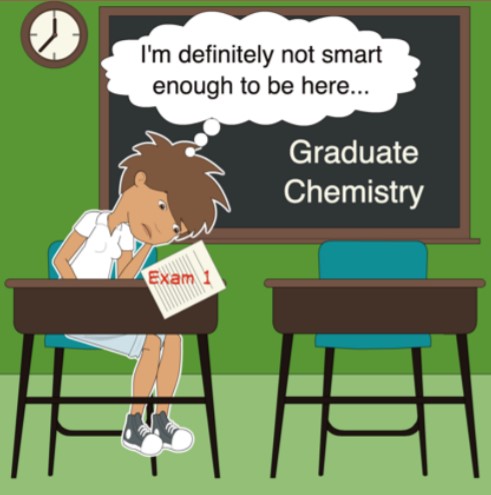


- 0 – Do not identify
- 1
- 2
- 3
- 4
- 5
- 6
- 7
- 8
- 9
- 10 - Very strongly identify

14.


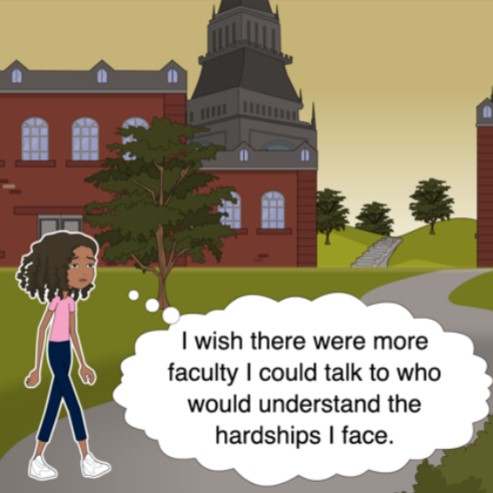


- 0 – Do not identify
- 1
- 2
- 3
- 4
- 5
- 6
- 7
- 8
- 9
- 10 - Very strongly identify

15. How strongly do you identify with the character on the left of the cartoon?


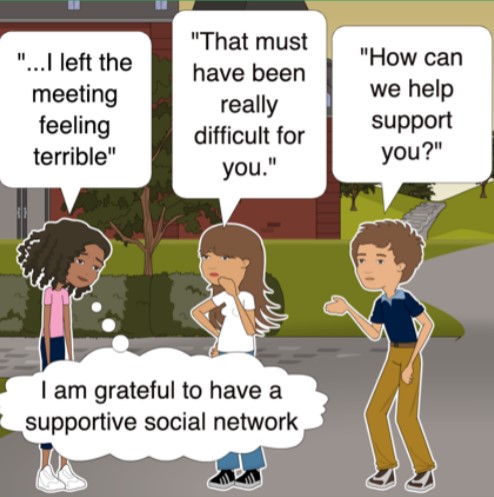


- 0 – Do not identify
- 1
- 2
- 3
- 4
- 5
- 6
- 7
- 8
- 9
- 10 - Very strongly identify

16.


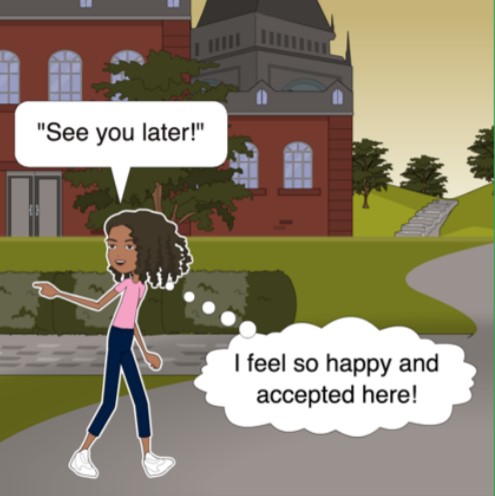


- 0 – Do not identify
- 1
- 2
- 3
- 4
- 5
- 6
- 7
- 8
- 9
- 10 - Very strongly identify

17.


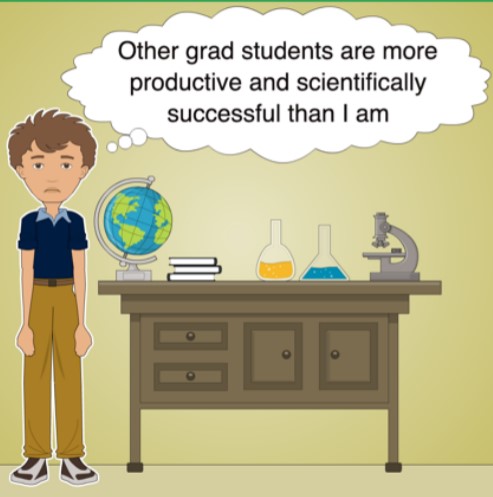


- 0 – Do not identify
- 1
- 2
- 3
- 4
- 5
- 6
- 7
- 8
- 9
- 10 - Very strongly identify

18.


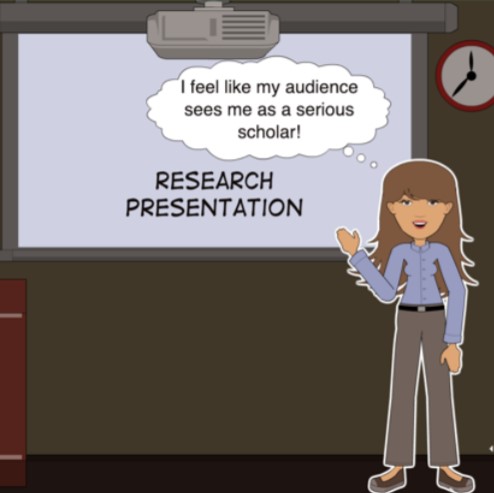


- 0 – Do not identify
- 1
- 2
- 3
- 4
- 5
- 6
- 7
- 8
- 9
- 10 - Very strongly identify

19.


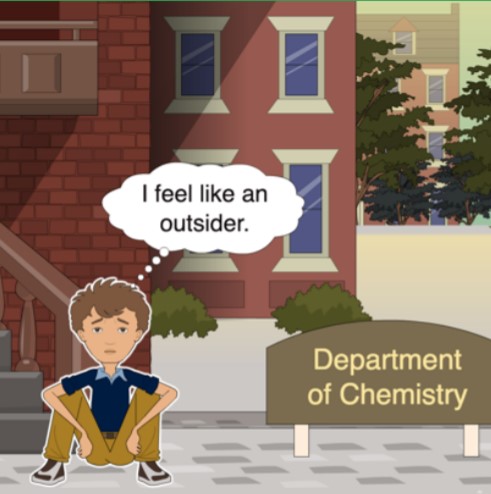


- 0 – Do not identify
- 1
- 2
- 3
- 4
- 5
- 6
- 7
- 8
- 9
- 10 - Very strongly identify

20.


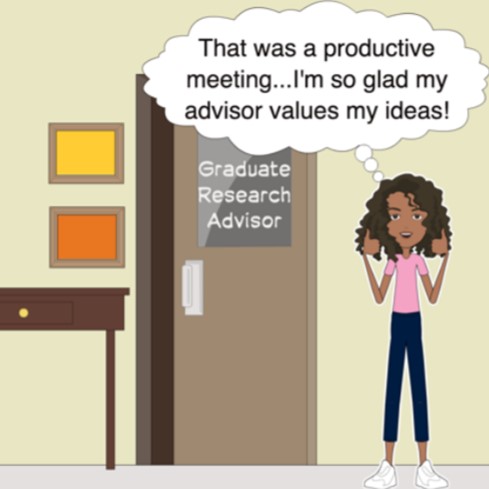


- 0 – Do not identify
- 1
- 2
- 3
- 4
- 5
- 6
- 7
- 8
- 9
- 10 - Very strongly identify

21.


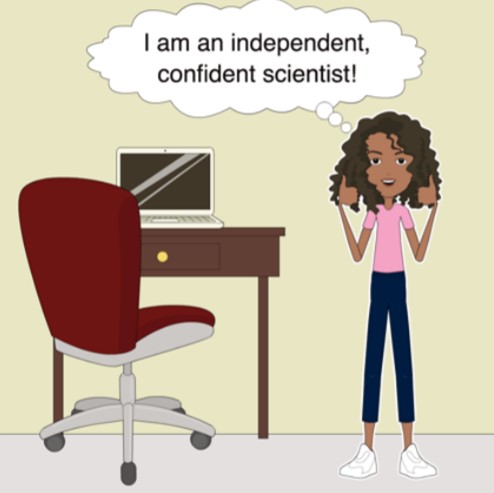


- 0 – Do not identify
- 1
- 2
- 3
- 4
- 5
- 6
- 7
- 8
- 9
- 10 - Very strongly identify

22. Think about what it means to you to feel "at home" somewhere. How does this feeling apply to your experience in the [Departments of Chemistry and Chemical and Biomolecular Engineering at this institution]?

________________________________________________________________

________________________________________________________________

________________________________________________________________

________________________________________________________________

________________________________________________________________

23. What to you constitutes a scientist? Do you describe yourself as a scientist? Why or why not? (For engineering students, please answer these questions for "engineer" rather than "scientist").

________________________________________________________________

________________________________________________________________

________________________________________________________________

________________________________________________________________

________________________________________________________________

Your data will be kept private and only accessible by the study directors via password-protected computers. Please keep in mind that while your data is kept as secure and private as possible, no guarantees can be made against the low risk of an inadvertent breach in confidentiality. For this reason, if there is any risk that a breach of your responses would lead to any damage or discomfort we encourage you to please leave the question blank.

24. (Optional) Please state the gender you best identify with.

________________________________________________________________

25. (Optional) Please state the sexuality you best identify with.

________________________________________________________________

26. (Optional) Please state the race/ethnicity you best identify with.

________________________________________________________________

27. (Optional) Are you the first in your immediate family to attend college?

- Yes
- No

TSMP Graduate Student Pre-Program Survey (August 2021)

All images used in this survey were reproduced under CC BY International 4.0 License, from ref 1. **2020**, *PLOS ONE*.

1. What year are you?

- 1st year
- 2nd year
- 3rd year
- 4th year
- 5th year
- 6th year
- Other ________________________________________________

2. Have you previously worked with the Transfer Student Mentorship Program (TSMP)?

- Yes
- No
- Other ________________________________________________

3. Please briefly describe any previous experience you've had mentoring (can simply list mentorship positions).

________________________________________________________________

4. How strongly do you identify with the character on the right in the cartoon?


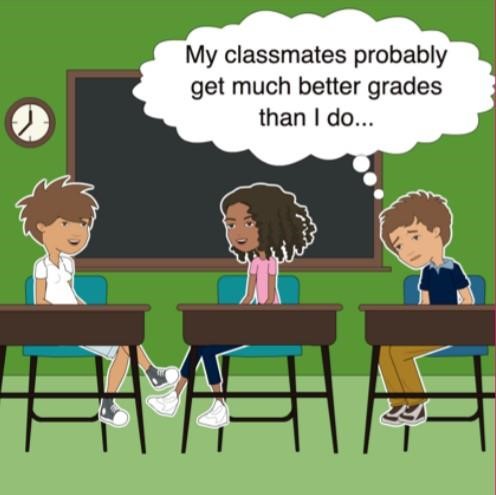


- 0 – Do not identify
- 1
- 2
- 3
- 4
- 5
- 6
- 7
- 8
- 9
- 10 - Very strongly identify

5.


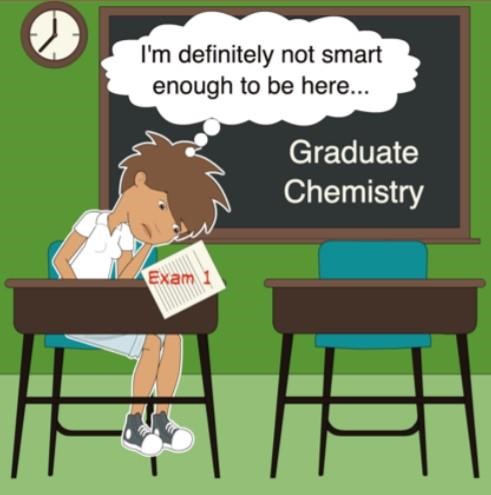


- 0 – Do not identify
- 1
- 2
- 3
- 4
- 5
- 6
- 7
- 8
- 9
- 10 - Very strongly identify

6.


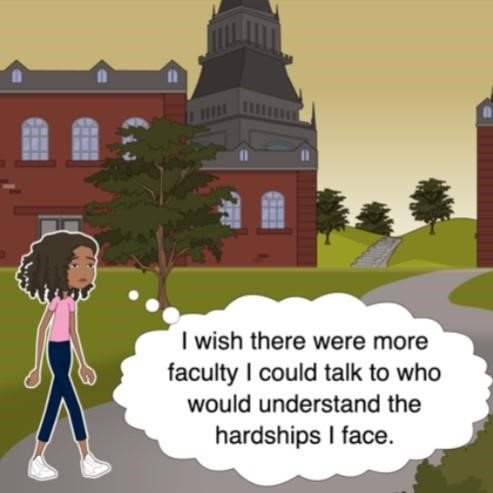


- 0 – Do not identify
- 1
- 2
- 3
- 4
- 5
- 6
- 7
- 8
- 9
- 10 - Very strongly identify

7. How strongly do you identify with the character on the left of the cartoon?


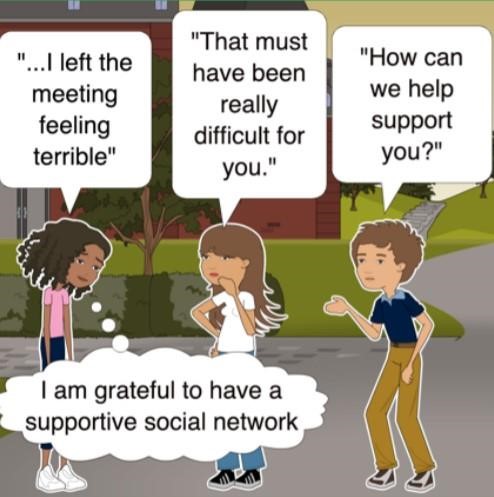


- 0 – Do not identify
- 1
- 2
- 3
- 4
- 5
- 6
- 7
- 8
- 9
- 10 - Very strongly identify

8.


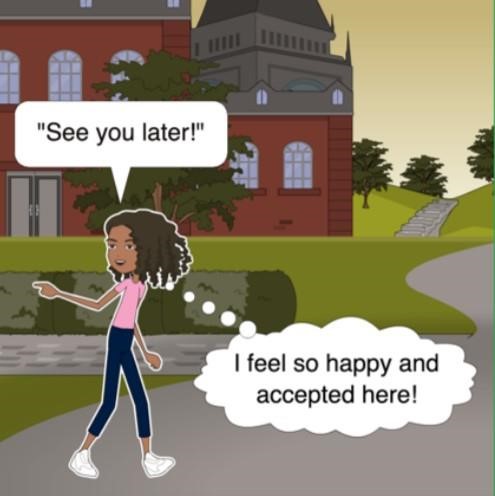


- 0 – Do not identify
- 1
- 2
- 3
- 4
- 5
- 6
- 7
- 8
- 9
- 10 - Very strongly identify

9.


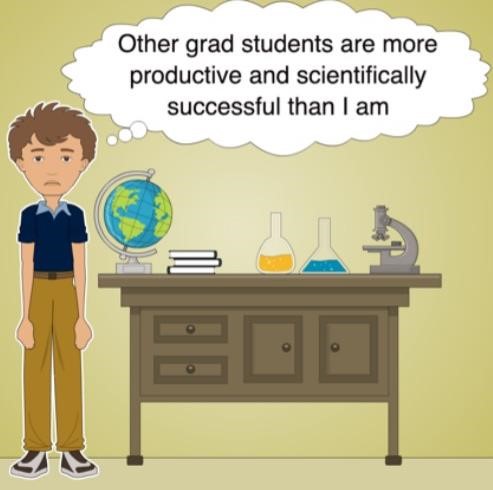


- 0 – Do not identify
- 1
- 2
- 3
- 4
- 5
- 6
- 7
- 8
- 9
- 10 - Very strongly identify

10.


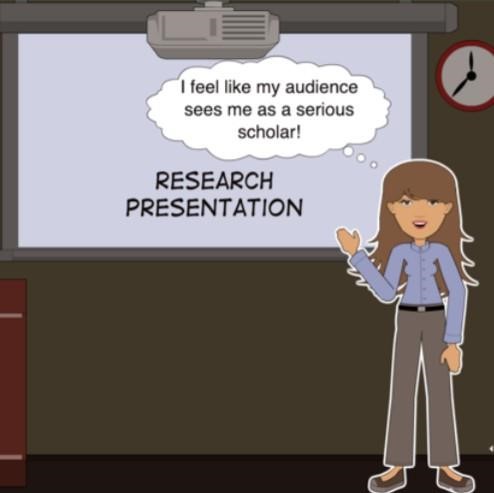


- 0 – Do not identify
- 1
- 2
- 3
- 4
- 5
- 6
- 7
- 8
- 9
- 10 - Very strongly identify

11.


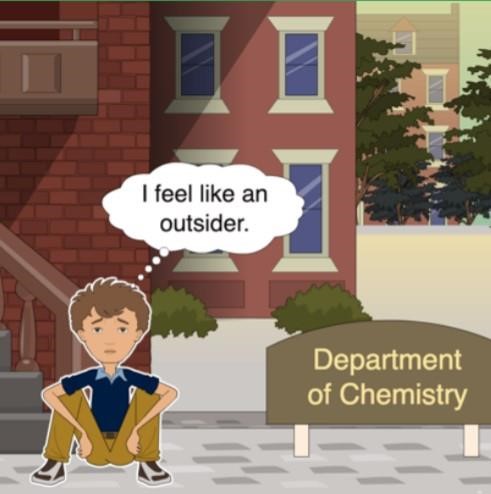


- 0 – Do not identify
- 1
- 2
- 3
- 4
- 5
- 6
- 7
- 8
- 9
- 10 - Very strongly identify

12.


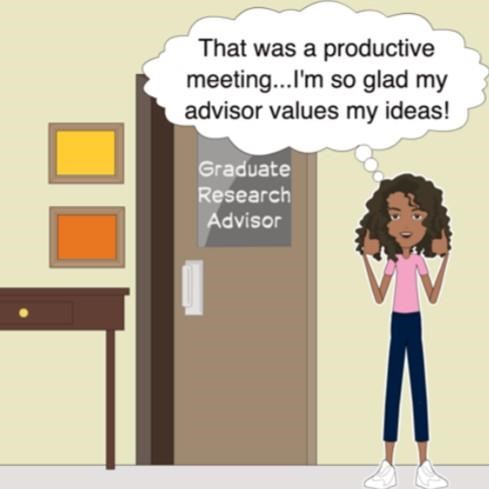


- 0 – Do not identify
- 1
- 2
- 3
- 4
- 5
- 6
- 7
- 8
- 9
- 10 - Very strongly identify

13.


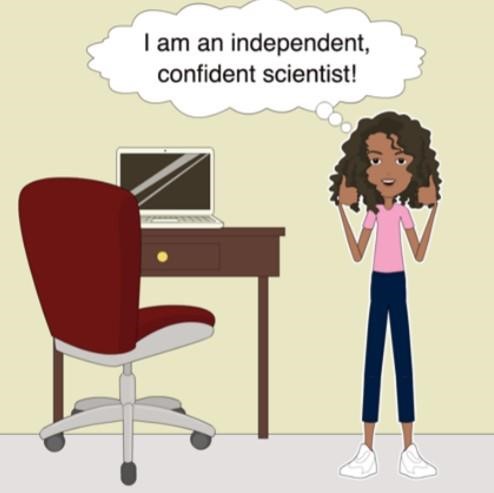


- 0 – Do not identify
- 1
- 2
- 3
- 4
- 5
- 6
- 7
- 8
- 9
- 10 - Very strongly identify

14. Think about what it means to you to feel "at home" somewhere. How does this feeling apply to your experience in the [Departments of Chemistry and Chemical and Biomolecular Engineering at this institution]?

________________________________________________________________

________________________________________________________________

________________________________________________________________

________________________________________________________________

________________________________________________________________

15. What to you constitutes a scientist? Do you describe yourself as a scientist? Why or why not?

________________________________________________________________

________________________________________________________________

________________________________________________________________

________________________________________________________________

________________________________________________________________

Your data will be kept private and only accessible by the study directors via password-protected computers. Please keep in mind that while your data is kept as secure and private as possible, no guarantees can be made against the low risk of an inadvertent breach in confidentiality. For this reason, if there is any risk that a breach of your responses would lead to any damage or discomfort we encourage you to please leave the question blank.

16. (Optional) Please state the gender you best identify with.

________________________________________________________________

17. (Optional) Please state the sexuality you best identify with.

________________________________________________________________

18. (Optional) Please state the race/ethnicity you best identify with.

________________________________________________________________

19. (Optional) Are you the first in your immediate family to attend college?

- Yes
- No

TSMP Graduate Student Post-Program Survey (December 2021)

All images used in this survey were reproduced under CC BY International 4.0 License, from ref 1. **2020**, *PLOS ONE*.

1. What year are you?

- 1st year
- 2nd year
- 3rd year
- 4th year
- 5th year
- 6th year
- Other ________________________________________________

2. Have you previously worked with the Transfer Student Mentorship Program (TSMP)?

- Yes
- No
- Other ________________________________________________

3. Please briefly describe any previous experience you've had mentoring (can simply list mentorship positions).

________________________________________________________________

4. Approximately how many times did you meet with your transfer student mentee?

________________________________________________________________

5. Did you feel that you and your transfer student mentee were well matched? Please outline why or why not.

________________________________________________________________

________________________________________________________________

________________________________________________________________

________________________________________________________________

________________________________________________________________

6. How many TSMP meetings did you attend?

- 1
- 2
- 3
- 4
- 5
- 6

7. Do you have an ongoing relationship with any other participants you met through the TSMP?

________________________________________________________________

8. Which TSMP seminar did you find the most useful and why?

________________________________________________________________

9. What has the impact of the TSMP been on your professional development?

________________________________________________________________

10. How strongly do you identify with the character on the right in the cartoon?


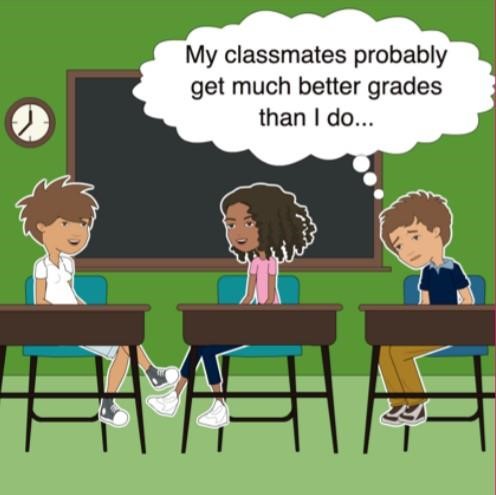


- 0 – Do not identify
- 1
- 2
- 3
- 4
- 5
- 6
- 7
- 8
- 9
- 10 - Very strongly identify

11.


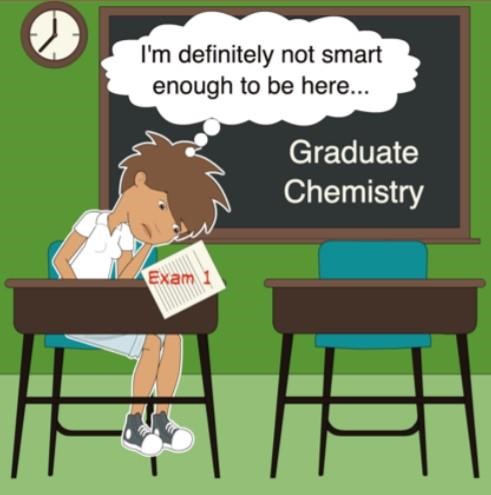


- 0 – Do not identify
- 1
- 2
- 3
- 4
- 5
- 6
- 7
- 8
- 9
- 10 - Very strongly identify

12.


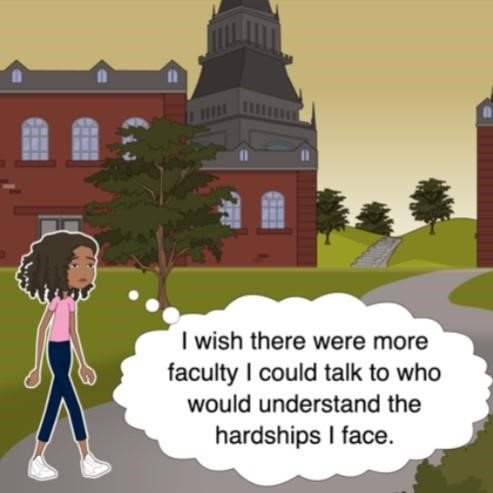


- 0 – Do not identify
- 1
- 2
- 3
- 4
- 5
- 6
- 7
- 8
- 9
- 10 - Very strongly identify

13. How strongly do you identify with the character on the left of the cartoon?


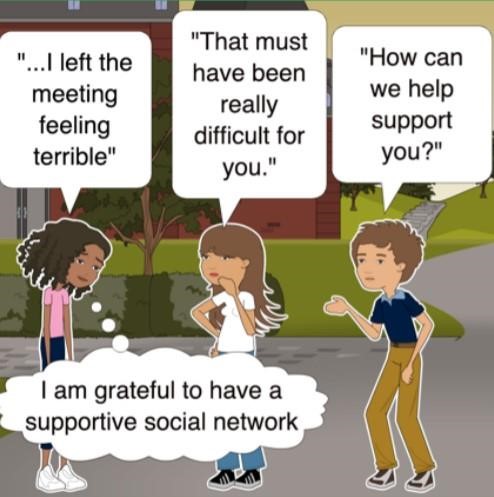


- 0 – Do not identify
- 1
- 2
- 3
- 4
- 5
- 6
- 7
- 8
- 9
- 10 - Very strongly identify

14.


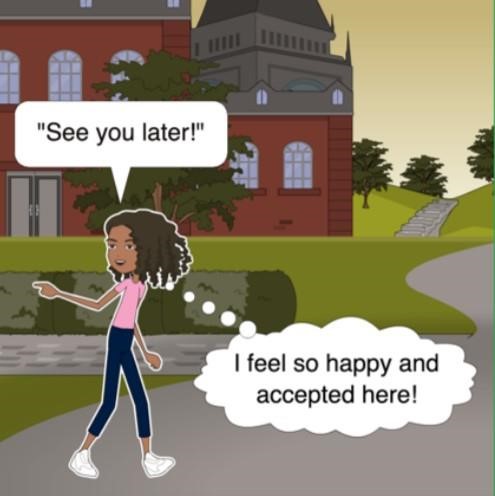


- 0 – Do not identify
- 1
- 2
- 3
- 4
- 5
- 6
- 7
- 8
- 9
- 10 - Very strongly identify

15.


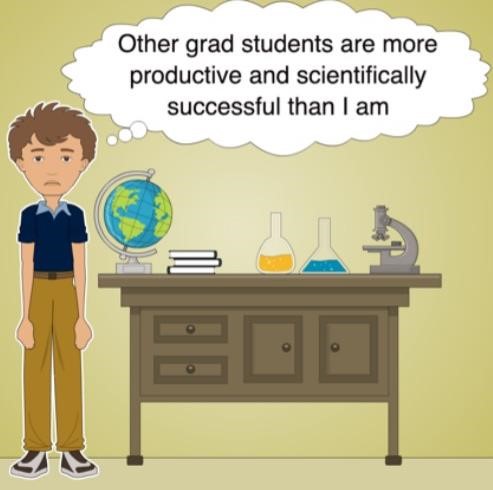


- 0 – Do not identify
- 1
- 2
- 3
- 4
- 5
- 6
- 7
- 8
- 9
- 10 - Very strongly identify

16.


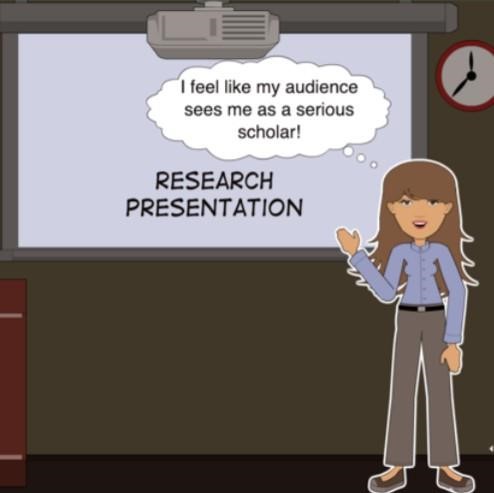


- 0 – Do not identify
- 1
- 2
- 3
- 4
- 5
- 6
- 7
- 8
- 9
- 10 - Very strongly identify

17.


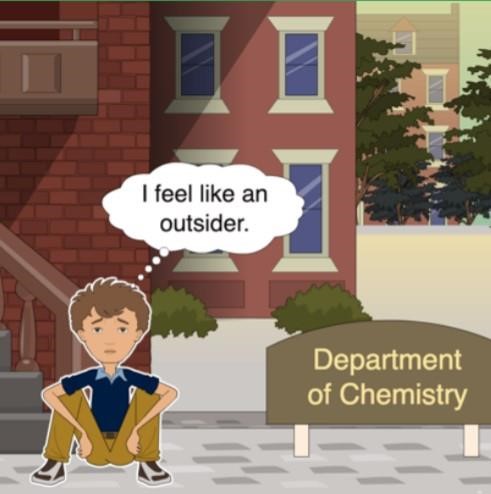


- 0 – Do not identify
- 1
- 2
- 3
- 4
- 5
- 6
- 7
- 8
- 9
- 10 - Very strongly identify

18.


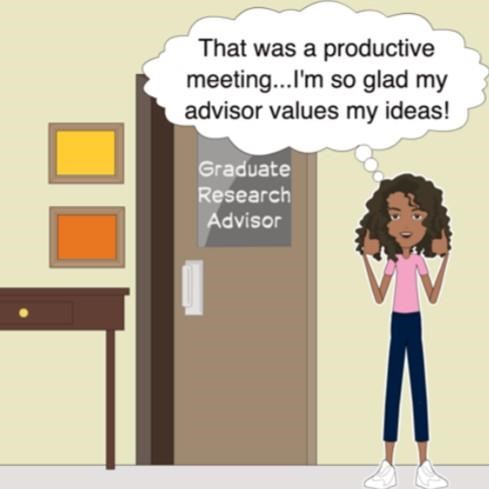


- 0 – Do not identify
- 1
- 2
- 3
- 4
- 5
- 6
- 7
- 8
- 9
- 10 - Very strongly identify

19.


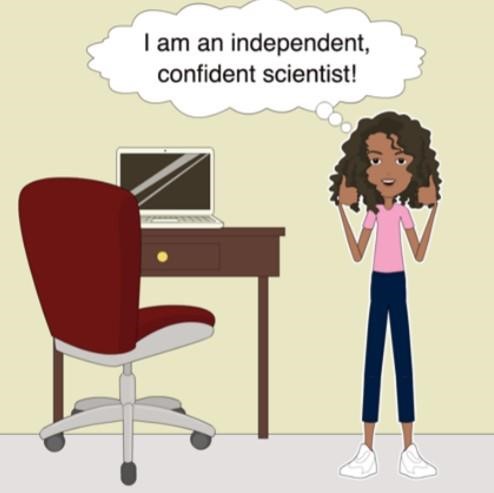


- 0 – Do not identify
- 1
- 2
- 3
- 4
- 5
- 6
- 7
- 8
- 9
- 10 - Very strongly identify

20. Think about what it means to you to feel "at home" somewhere. How does this feeling apply to your experience in the [Departments of Chemistry and Chemical and Biomolecular Engineering at this institution]?

________________________________________________________________

________________________________________________________________

________________________________________________________________

________________________________________________________________

________________________________________________________________

21. What to you constitutes a scientist? Do you describe yourself as a scientist? Why or why not?

________________________________________________________________

________________________________________________________________

________________________________________________________________

________________________________________________________________

________________________________________________________________

Your data will be kept private and only accessible by the study directors via password-protected computers. Please keep in mind that while your data is kept as secure and private as possible, no guarantees can be made against the low risk of an inadvertent breach in confidentiality. For this reason, if there is any risk that a breach of your responses would lead to any damage or discomfort we encourage you to please leave the question blank.

22. (Optional) Please state the gender you best identify with.

________________________________________________________________

23. (Optional) Please state the sexuality you best identify with.

________________________________________________________________

24. (Optional) Please state the race/ethnicity you best identify with.

________________________________________________________________

25 (Optional) Are you the first in your immediate family to attend college?

- Yes
- No

**TSMP Graduate Student Follow-Up Survey (March 2022)**

1. To the best of your knowledge, did your first mentee have a research position before the beginning of the program?

- Yes
- No
- I don't know
- Other________________________________________________

2. To the best of your knowledge, does your first mentee have a research position now?

- Yes
- No
- I don't know
- Other________________________________________________

3. Approximately how many times did you meet with your first mentee?

- 0
- 1
- 2
- 3
- 4
- 5
- 6
- Other________________________________________________

The following questions are for mentors who had more than one mentee. If you had only one mentee, please skip to the end of the survey and click the forward arrow to finish.

4. To the best of your knowledge, if you had a second mentee, did your second mentee have a research position before the beginning of the program?

- Yes
- No
- I don't know
- Other________________________________________________

5. To the best of your knowledge, if you had a second mentee, does your second mentee have a research position now?

- Yes
- No
- I don't know
- Other________________________________________________

6. If you had a second mentee, approximately how many times did you meet with your second mentee?

- 0
- 1
- 2
- 3
- 4
- 5
- 6
- Other________________________________________________

**References**

1. Stachl, C. N.; Baranger, A. M. Sense of Belonging within the Graduate Community of a Research-Focused STEM Department: Quantitative Assessment Using a Visual Narrative and Item Response Theory. *PLOS ONE* **2020**, *15 (5)*, 1–27. DOI: 10.1371/journal.pone.0233431.
